# Disinhibition of ventral tegmental area during initial punishment learning causes enduring punishment insensitivity

**DOI:** 10.1038/s41386-026-02368-4

**Published:** 2026-02-17

**Authors:** Shannen Y. S. Tan, Michelle H. Shen, Luke J. Keevers, Matthew Williams-Spooner, Gavan P. McNally, Simon Killcross, Philip Jean-Richard-dit-Bressel

**Affiliations:** School of Psychology, UNSW, Sydney, Australia

**Keywords:** Operant learning, Neurophysiology, Risk factors

## Abstract

Avoiding actions with negative consequences is fundamental to adaptive behavior. Traditional theories suggest GABAergic inhibition of midbrain dopamine neurons, including those within ventral tegmental area (VTA_DA_), mediate suppression of actions that lead to aversive outcomes. However, the role of dopamine inhibition in punishment learning remains unclear. To examine this, we conducted fiber photometry, pharmacological, and chemogenetic experiments in rats to measure VTA_DA_ activity and GABA input across punishment learning, and test their causal contribution to behavior. VTA_DA_ activity and GABA input phasically increased to response-elicited outcomes, with VTA_DA_ activity being more strongly activated by rewards, while GABA input being more strongly activated by shock punishers during initial punishment. Pharmacologically blocking GABA_A_ receptors in VTA or chemogenetically activating VTA_DA_ neurons during initial, but not later, punishment sessions produced enduring deficits in punishment avoidance. These findings suggest long-term avoidance depends upon a critical window of GABA-mediated VTA_DA_ inhibition during punishment learning.

## Introduction

Actions with positive consequences tend to be reinforced (i.e., repeated), whereas actions with negative consequences tend to be punished (i.e., suppressed) [1]. This fundamental adaptive function, known as instrumental conditioning, helps organisms dynamically adjust their behavior to maximize rewards and minimize harms.

Dopaminergic neurons of the ventral midbrain, including those within the ventral tegmental area (VTA_DA_), are widely considered to be critical for this learning [2–6]. These neurons exhibit phasic increases in activity to better-than-expected rewards, which are considered necessary and sufficient for reinforcing behaviors [7–10]. Conversely, VTA_DA_ neurons exhibit phasic decreases in activity to worse-than-expected outcomes (including aversive events) [5, 11, 12], and inhibition of VTA_DA_ has been shown to be aversive [13–15].

Crucially, when specific actions cause brief optogenetic inhibition of VTA_DA_ neurons, those actions (but not other actions) are suppressed [13]. This shows pauses in VTA_DA_ neuron activity can function as punishment to produce selective instrumental avoidance. Endogenously, pauses in VTA_DA_ activity are driven by GABA input to VTA_DA_ neurons from local interneurons and long-range GABA inputs [5, 16, 17], which act on GABA_A_ receptors to suppress VTA_DA_ firing [15, 18, 19]. However, patterns of GABA release onto VTA_DA_ neurons during punishment and the necessity of VTA_DA_ inhibition in punishment avoidance remain unclear.

To examine this, we performed fiber photometry recordings of VTA_DA_ calcium (Ca^2+^; neural activity proxy) and GABA input across a punishment task to characterize how VTA_DA_ and GABA dynamics relate to behavior under punishment. We tested the causal significance of VTA GABA input and VTA_DA_ activity on punishment learning and choice via pharmacological (GABA_A_ receptor antagonist) and chemogenetic manipulations (hM3D DREADD) across phases of punishment, and show preventing GABA_A_-mediated inhibition of VTA and direct excitation of VTA_DA_ during initial punishment learning produces enduring punishment insensitivity.

## Methods

Further details for Methods are supplied in **Supplemental Materials**.

### Subjects

All experiments used experimentally-naive rats aged 8-24 weeks old. Photometry and chemogenetic experiments used heterozygous TH::Cre Sprague Dawley rats (SD-Th-cre^tm1sage^; Sage Laboratories). TH::Cre+ animals express Cre in tyrosine hydroxylase (TH; dopamine precursor enzyme) neurons; TH::Cre- animals (used in control experiment reported in Supplemental Materials) do not express Cre. Experiment 2 (pharmacological manipulation) used wild-type Sprague Dawley rats. Subjects across experiments were males, except for Experiment 1 GABA recordings, which included both male and female subjects. Where applicable, we report data by sex in **Supplemental Materials**, including a supplemental behavioral study comparing males and females (Fig. S1).

Animals were group-housed (4) in plastic cages in a climate-controlled colony room maintained on a 12 h light–dark cycle. Rats had *ad libitum* access to chow until 2 days before behavioral training, after which they received 10–15 g chow daily (after behavioral session) to maintain them at ~90% of their free-feeding weight. Rats had access to water in their homecages throughout experiments. All procedures were approved by the Animal Care and Ethics Committee at UNSW Sydney and conducted in accordance with the National Health and Medical Research Council Code for the Care and Use of Animals for Scientific Purposes in Australia (2013).

### Apparatus & materials

All operant behavior was assessed in MedAssociates operant chambers, each housed within light and sound-attenuating cabinets. Each chamber contained two retractable levers that flanked a magazine port where grain pellet rewards were delivered. The punisher was a 0.5 s footshock, delivered through the grid floor. Footshock intensity was 0.4 mA for fiber photometry experiments, and 0.5 mA for neural manipulation experiments. A lower intensity footshock was chosen for photometry experiments to avoid floor effects in responding that would undermine key analyses of peri-event dynamics.

Locomotor tests were conducted in open field chambers that tracked movement via 16-beam infrared arrays located along X- and Y-axes.

Fiber photometry recordings were conducted using Doric Lenses photometry components (465 nm and 405 nm LEDs, mini-cubes, photodetectors) and Tucker Davis Technologies photometry processor (RZ5P).

Adeno-associated viruses (AAVs) were used to express Cre-dependent calcium sensor (AAV-CAG-DIO-GCaMP6f), GABA sensor (AAV-hSyn-DIO-iGABASnFR-F102G), or excitatory DREADD (AAV-hSyn-DIO-hM3D-mCherry) in VTA_DA_ neurons of TH::Cre+ animals.

Microinfusions of GABA_A_ antagonist bicuculline (0.1 μg/μl; Tocris, Sydney, Australia) were used to prevent GABA-mediated inhibition in VTA [15, 18, 19]. Systemic injections of 3 mg/kg clozapine-*N*-oxide (CNO; National Institute of Mental Health Chemical Synthesis and Drug Supply Program), dissolved in 5% DMSO and saline, were used to activate hM3D. Potential off-target effects of CNO [20] were addressed via TH::Cre- control subjects (**Supplemental Materials**–Fig. S4).

### Surgeries

Rats were anaesthetized and placed into a flat skull position within a stereotaxic frame. Craniotomies were performed above VTA. For photometry and chemogenetic experiments, a 5 μl 30-gauge microinfusion syringe (Hamilton; Reno, NV, USA) was used to inject 0.75 μl AAVs (0.25 μl/min) encoding Cre-dependent GCaMP6f (unilateral), iGABASnFR (unilateral), or hM3D (bilateral) into VTA (AP: -5.5, ML: ±0.8, DV: -8.2 from bregma) of TH::Cre rats. Following injections, the syringe remained at the injection site for an additional 5 min for diffusion.

For photometry experiments, a 400μm optic fiber was unilaterally implanted into VTA (AP: -5.5, ML: ±0.8, DV: -8.2 from bregma). For pharmacology experiments, a bilateral 26-gauge 11 mm guide cannula (PlasticsOne) was implanted into VTA (AP: -5.8, ML: ±0.75, DV: -8.2 from bregma). Implants were anchored in position with dental cement and jeweller’s screws. Immediately following surgery, animals were given antibiotics and received post-operative monitoring and care for 1 week. Rats that received AAV injections were given an additional 3 weeks before behavioral training to allow sufficient transgene expression.

### Behavioral task

All rats underwent a previously validated punishment task, which has been shown to elicit robust punishment avoidance with minimal contamination from Pavlovian fear [21–23].

#### Lever-press training

Rats were first trained to press two levers (R1, R2) for food. For 2 sessions, both levers were presented concurrently, and each press on a lever was rewarded with a pellet (FR1 training). A lever remained extended until it received 25 presses or after 1 h. Rats that failed to acquire lever-pressing were manually shaped in the second FR1 session.

Rats then received 7-8 days of VI30s training (40 min sessions). In these sessions, levers were presented individually for 5 min blocks in alternating fashion (first lever randomized per day). Lever-presses were reinforced on a 30 s variable interval (VI30s) schedule, such that the first press after an average interval of 30 seconds led to pellet delivery.

#### Punishment

Subjects then received daily 40 min punishment sessions. Lever-pressing on either lever continued to yield pellets (VI30s). However, every 10^th^ press (FR10) on the punished R1 lever resulted in immediate footshock delivery. Presses on the unpunished R2 lever had no additional consequence. If a press was scheduled to deliver both footshock and pellet, both were delivered. Assignment of left vs. right levers as punished vs. unpunished was counterbalanced across (but not within) subjects.

For pharmacology experiments, rats received intra-VTA infusions of 0.5 µl GABA_A_ antagonist bicuculline or control saline (0.25 µl/min; 1 min diffusion) immediately prior to the first two sessions of punishment (between-subjects), and bicuculline vs. saline on punishment days 6 and 7 (within-subjects, order counterbalanced). This design permits efficient interrogation of neural manipulation effects on acquisition and expression of punishment avoidance [21, 22].

The same design was employed for DREADD manipulations, except rats received i.p. injections of CNO or vehicle (30 min before session start) instead of microinfusions, and expression tests were conducted on punishment days 7 and 8.

#### Choice test

Rats were then given choice test(s) where both levers were presented concurrently. No shocks were delivered and presses on either lever delivered pellets on a shared VI60s schedule, so there was no advantage to pressing either lever exclusively or a combination of both levers.

Photometry experiments only involved a single 15 min choice test. For manipulation experiments, animals received within-subjects drug vs. control across two choice tests (order counterbalanced) (pharmacology experiment: 30 min tests; DREADD experiment: 20 min tests). Each choice test was preceded by a drug-free punishment session the day prior [21, 22].

### Locomotor tests

Effects of VTA manipulation on locomotion were assessed following completion of the punishment task. Rats first received a 30 min habituation session, where they were placed into the open field chamber without any injections. On the following 2 days, rats received drug or control injection (within-subjects, counterbalanced order) before being placed into the chambers for 30 min to assess distance traveled.

### Histology

At the end of all experiments, brain tissue was examined to verify virus expression and/or implant locations. For photometry and chemogenetic experiments, animals were anaesthetized and perfused with 4% paraformaldehyde. Fixed brains were sectioned using a cryostat. Virus expression was determined via immunohistochemistry using anti-GFP (biosensors) and anti-TH (tyrosine hydroxylase [dopamine neuron marker]) primary antibodies and fluorescent secondary antibodies. For pharmacology experiments, animals were euthanized and unfixed brains were sectioned using a cryostat, slide-mounted, and stained with cresyl violet.

### Data analysis

Rats that failed to acquire lever-pressing during lever training, or had inappropriate virus expression or implant placements, were excluded from all analyses.

#### Behavior analysis

The key behavioral dependent measures were self-normalized rates of responding on each lever (“suppression ratios”) [23], and average latency to initially press each lever across trials (averaged per session).

Suppression ratios normalize response rates per lever during punishment and choice sessions to pre-punishment (final VI30s) rates. This was calculated per lever as follows:

$${Suppression}\,{ratio}=\frac{{Session}\,{LP}\,{rate}}{({Session}\,{LP}\,{rate}+{Training}\,{LP}\,{rate})}$$

Suppression ratios can range from 0 to 1. Scores above 0.5 indicate greater lever-pressing relative to training, scores below 0.5 indicate less lever-pressing, while a score of 0.5 indicates no difference relative training. This was done to address any spurious difference in punished or unpunished response rates prior to punishment. Nonetheless, analyses of raw response rates are provided in Supplemental Materials.

Behavioral data was analyzed using repeated measures ANOVA. Within-subjects factors were lever, session, and drug. Between-subjects factor was acquisition group (drug vs. control). For all analyses, Type 1 error was controlled at 0.05.

#### Fiber photometry analysis

465 nm (neural dynamic-related) and 405 nm (isosbestic control) signals and event timestamps were extracted into MATLAB, and signals during logged disconnections were discarded. Each signal was low-pass (3 Hz) and notch (1.0322-1.0326, 2.547-2.55 Hz) filtered to remove high-frequency noise identified via Fast Fourier Transform. Filtered 405 nm signals were fit to filtered 465 nm signals via iteratively-reweighted least squares [24] to create fitted 405 nm signals. A normalized fluorescence change score (dF/F) was calculated using the standard formula:

$${dF}/F=\frac{(465{nm}\,{signal}-{fitted}\,405{nm})}{{fitted}\,405{nm}}$$

This motion-artifact-corrected dF/F was detrended via 60 s moving median (5 s mean smoothing window) and converted into standard deviation units by dividing session signals by their sum squared deviation from 0 (nullZ-score) [24]. All photometry analyses were derived from this normalized, artifact-corrected dF/F.

The key dependent variable was change in VTA_DA_ activity and GABA input around response-elicited outcomes (reward delivery, footshock) and actions (R1, R2). dF/F around pellets vs. footshocks, and R1 vs. R2 lever-presses alone (i.e., those not yielding footshock or pellets) were collated. Each trial was re-zeroed to pre-event baseline (-5:-3 s) and averaged per subject; all analyses used mean peri-event transients per subject. Due to the scarcity of punished lever-presses and footshocks in late punishment sessions, late punishment data (Pun4 onwards) was combined to obtain more accurate peri-event activity traces per subject, as done for previous studies [23]. Significant transients were identified via bootstrapped confidence intervals (CI) [25]. Bootstrapped means were obtained by randomly resampling from subject mean waveforms with replacement (1000 iterations). 95% CI limits were derived from 2.5 and 97.5 percentiles of bootstrap distribution, expanded by a factor of √(n/(n-1)). A significant transient was identified as a period that CI limits did not contain 0 (pre-event baseline) for at least 1/3 s (low-pass filter window [25]). Significant differences between event waveforms were similarly determined by bootstrapping the within-subject difference waveform (mean event1–mean event2 waveform) per subject [25].

Additional analysis methods applying correlations and General Linear Modeling to photometry data are reported in **Supplemental Materials**.

## Results

### Experiment 1: VTA_DA_ neuron activity and GABA input during punishment learning

**Fig. 1:**
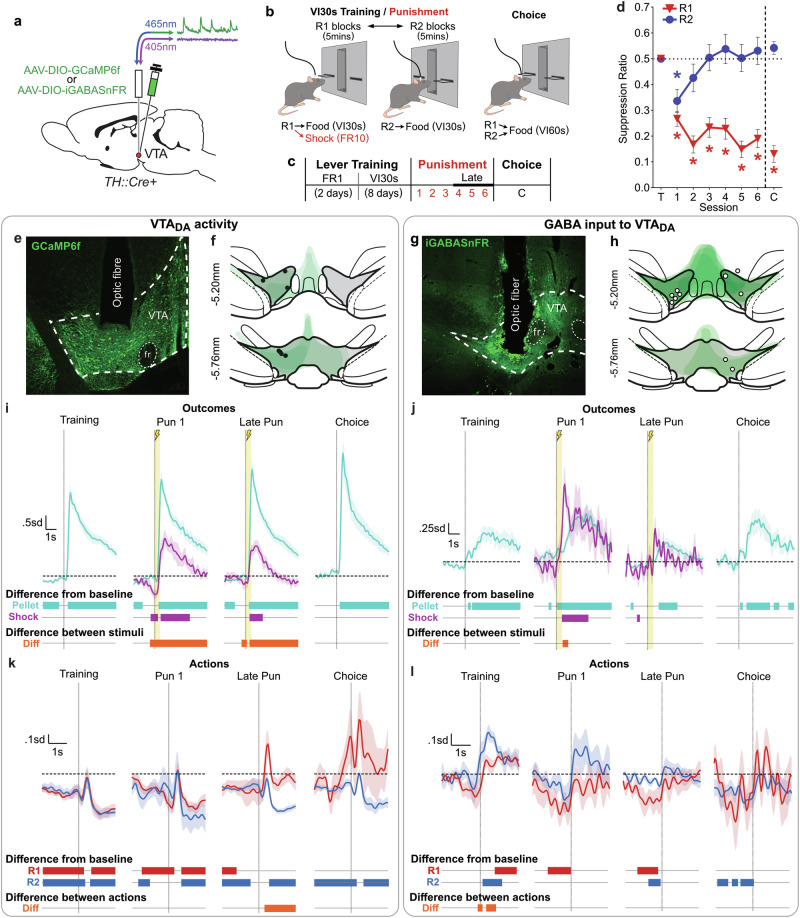
VTA_DA_ activity and GABA input during punishment learning. **a** TH::Cre+ rats received Cre-dependent calcium or GABA sensors and fiber implant into VTA. **b** Punishment task. Rats could press two levers (R1, R2 [5 min alternating blocks]) for food. In punishment sessions, R1 responses also yielded footshock (FR10 schedule). During choice test, both levers were presented to assess lever preference. **c** Timeline of task sessions. **d** Mean ± SEM lever-press ratios for the last session of lever training (T), punishment sessions (1-6) and choice (C) across subjects (*N* = 14). Punishment led to robust, selective suppression of R1 responding, and a strong preference for unpunished R2 over previously-punished R1 during choice test. **e** Example GCaMP expression and fiber placement. **f** Placement map for rats with valid GCaMP and fiber tip locations (*n* = 7). **g** Example iGABASnFR expression and fiber placement. **h** Placement map for rats with valid iGABASnFR and fiber tip locations (*n* = 10). **i, j** Mean ± SEM of subject-averaged VTA GCaMP **I** and iGABASnFR **J** signals around response-elicited appetitive and aversive outcomes (pellet delivery [teal] vs. footshock [purple]) during lever training, early punishment (Pun 1 [1^st^ session]), late punishment (sessions 4 + ), and choice sessions. Vertical dashed lines indicate event onset (yellow highlighted area indicates shock duration). Horizontal dotted line indicates pre-event baseline. Bars at the bottom indicate when peri-event signals significantly deviated from baseline, and when pellet-related and shock-related signals significantly differed from each other (orange bars). Appetitive and aversive events elicited increases in VTA_DA_ activity and GABA input, but GABA input tended to be greater for aversive events, while increases in VTA_DA_ activity were greater for rewards. **k, l** Mean ± SEM of subject-averaged VTA GCaMP **k** and iGABASnFR **l** signals around R1 vs. R2 actions (action alone; no outcomes delivered) for lever training, early punishment (Pun 1), late punishment, and choice sessions. Vertical dashed lines indicate time of lever-press. Bars at the bottom of each panel indicate when action-related signals significantly deviated from pre-event baseline (horizontal dashed line), and when action signals significantly differed from each other (orange bars). VTA_DA_ activity to punished R1 actions became dissociated from unpunished R2 actions as punishment was learned. GABA input to VTA_DA_ did not significantly distinguish between actions across punishment or choice.

We first examined activity of VTA_DA_ neurons and GABA input to VTA_DA_ neurons across punishment learning and choice (Fig. 1). This was done by selectively expressing GCaMP6f (Ca^2+^ sensor; neural activity proxy) or iGABASnFR (GABA sensor; GABA input proxy) in VTA_DA_ neurons of TH::Cre+ rats (Fig. S3) and recording from VTA across a punishment task (Fig. 1a–c). *N* = 14 rats had valid biosensor expression and fiber placements (*n* = 7 GCaMP [all males] [Fig. 1f]; *n* = 10 iGABASnFR [7 females] [Fig. 1h]) and were thus included in analyses. Male versus female data is shown in **Supplemental Materials**; there were no notable sex differences in behavior or GABA signals.

#### Task behavior

Animals first received lever-press training, where they could press two individually-presented levers (R1, R2) for food (Fig. 1b). Across this training, rats acquired similarly high rates of responding on R1 and R2 (lever: *F*_(1,12)_ = 0.10, *p* = .763); this did not depend on which sensor animals expressed (group: *F*_(1,12)_ = 1.19, *p* = .297; group*lever: *F*_(1,12)_ < 0.01, *p* = .949) (Fig. S2).

Rats then received punishment sessions, where lever-presses on R1 and R2 continued to yield food, but every 10^th^ press on R1 was punished with footshock (Fig. 1b). Rats were sensitive to this punishment schedule, selectively suppressing punished R1 responding relative to unpunished R2 (lever*: F*_(1,12)_ = 46.40, *p* < 0.001; group: *F*_(1,12)_ = 0.92, *p* = 0.357) (Fig. 1d). When given a choice test, where both levers were presented together and no shocks were delivered, rats showed a strong preference for the unpunished lever (lever: *F*_(1,12)_ = 65.71, *p* < 0.001; group: *F*_(1,12)_ = 0.04, *p* = 0.843) (Fig. 1d).

#### VTA_DA_ neural dynamics around appetitive and aversive outcomes

When examining activity of VTA_DA_ neurons around response-elicited outcomes, VTA_DA_ neurons exhibited pronounced excitatory Ca^2+^ transients to reward deliveries across sessions (95% CI > 0: ~0.4s onwards) (Fig. 1i). More surprisingly, excitatory transients were also observed to the shock punisher across punishment sessions. Critically, this excitatory shock transient began during shock delivery, and not simply to shock offset (Pun1 95% CI > 0 from 0.5–3.30 s relative to shock onset [not factoring ~0.2 s risetime for GCaMP6f [26]]; Late Pun 95% CI > 0 from 0.4–1.60 s). This contradicts canonical accounts of VTA_DA_ as reward coding, but is consistent with existing reports of some VTA_DA_ subpopulations being excited by aversive events [5, 27, 28].

There were also outcome-related fluctuations in GABA input to VTA_DA_ neurons (Fig. 1j). There were significant increases in GABA following reward deliveries. We also observed a sharp increase in GABA input in response to shocks during initial punishment sessions (Pun1 95% CI > 0 from 0.5–2.95s relative to shock onset [not factoring ~0.1 s risetime for iGABASnFR [29]]). By contrast, shock-related GABA transients during later punishment sessions did not significantly deviate from pre-event baseline. Interestingly, in contrast to VTA_DA_ neuron activity, phasic GABA signals were greater to shock than to reward. These observations conform with the idea that GABA input to VTA_DA_ provides a negative prediction error signal, suppressing VTA_DA_ neuron activity during expected rewards (i.e., rewards cued by the sound of pellet delivery) and unexpected aversive events [5, 15, 17].

It is worth noting here that GABA and VTA_DA_ signals were partly dissociated, as VTA_DA_ signals were not entirely suppressed during increased GABA input, and changes to shock-related GABA signaling was not paralleled by changes to VTA_DA_ signals to shock. This highlights independent excitatory input to VTA_DA_ and/or VTA_DA_ subpopulations that do not receive this increased GABA input [16].

#### VTA_DA_ neural dynamics around punished versus unpunished actions

To examine whether VTA neural dynamics tracked changing action values under punishment, we examined signals around punished vs. unpunished actions alone (i.e., actions not coinciding with outcome deliveries).

VTA_DA_ neurons exhibited punishment-related changes to activity around actions (Fig. 1k). Prior to punishment, VTA_DA_ neurons exhibited transient reductions in activity around each action relative to pre-event baseline. As punishment was learned, punished actions began eliciting excitatory transients, as previously reported [30], whereas unpunished actions retained their inhibitory activity pattern across punishment sessions.

Generally, we observed modest decreases in GABA signal in the lead up to actions (Fig. 1l). Besides a modest unexpected difference in GABA signal to punished versus unpunished actions in training, GABA release around punished versus unpunished actions were not significantly distinguished across punishment and choice.

Altogether, these findings indicate task-relevant fluctuations in VTA_DA_ population activity and GABA input to VTA_DA_. In partial agreement with traditional reward prediction error accounts, VTA_DA_ neurons were more strongly activated by rewards than aversive events, while GABA inputs to VTA_DA_ were more pronounced to aversive events. These dissociated dynamics to motivationally relevant events are thought to contribute to the reinforcing vs. punishing effects of outcomes on antecedent actions.

##### Experiment 2: Effects of GABA_A_ blockade in VTA during punishment

**Fig. 2:**
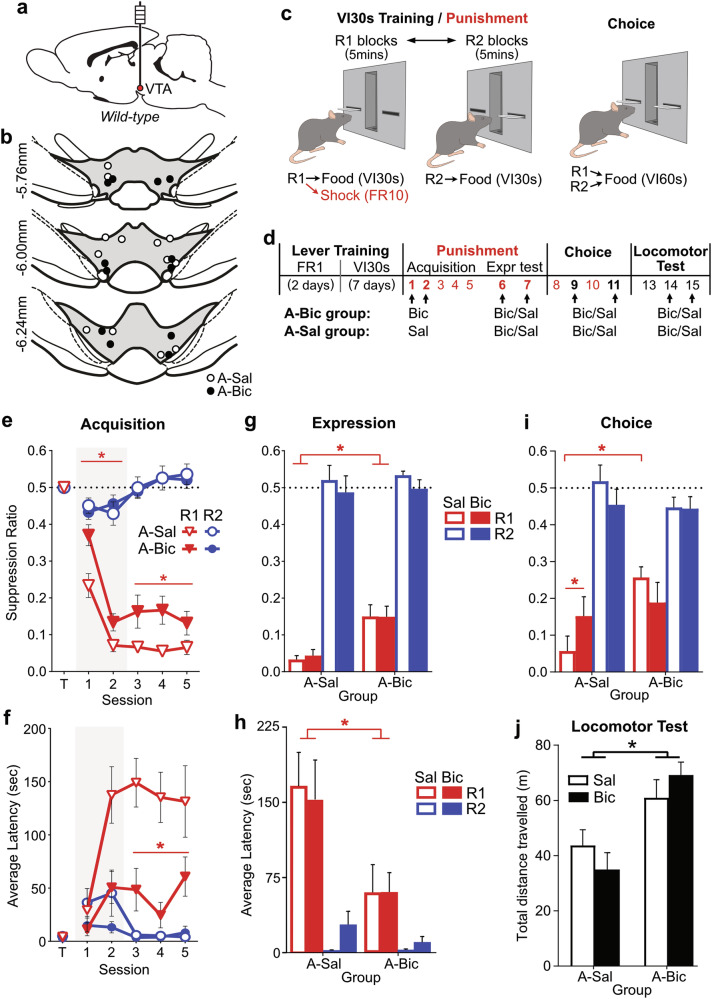
Effect of VTA GABA_A_ blockade on punished behavior. **a** Bilateral guide cannulae were implanted into VTA of wild-type rats **b** Cannulae placements for animals with valid placements, according to acquisition drug group (A-Sal [*n* = 7], A-Bic [*n* = 6]). **c** Punishment task design. **d** Timeline of task sessions, with arrows indicating when subjects received intra-VTA infusions of GABA_A_ antagonist bicuculline (Bic) and/or control saline (Sal). A-Bic vs. A-Sal groups received Bic vs. Sal (respectively) before the first 2 sessions of punishment. All groups received Bic vs. Sal (within-subjects, order counterbalanced) in subsequent punishment expression, choice, and locomotor tests. **e** Mean ± SEM lever suppression ratios by acquisition group across last day of training (T) and punishment acquisition sessions. Grey shaded area indicates infusion sessions. Rats that received Bic (A-Bic) exhibited a persistent deficit in punishment avoidance. **f** Mean ± SEM lever-press latencies by acquisition group across last day of training (T) and punishment acquisition sessions. A-Bic rats were quicker to press the punished lever relative to A-Sal rats. **g** Mean ± SEM lever suppression ratios during punishment expression tests per acquisition group. A-Bic rats continued to press punished R1 more than A-Sal rats; Bic infusions during expression tests had no effect on R1 responding. **h** Mean ± SEM lever-press latencies during punishment expression tests. A-Bic rats continued to press punished R1 more quickly than A-Sal rats; Bic infusions during expression tests had no effect on R1 responding. **i** Mean ± SEM suppression ratios during choice tests. Bic selectively increased R1 responding in A-Sal group. **j** Mean ± SEM distance traveled during locomotor tests. A-Bic rats traveled further than A-Sal animals. Bic infusions during these tests had no significant effect on this. *p < 0.05.

To examine the causal role GABA signaling within VTA on punishment, we implanted bilateral guide cannulae into VTA of male wild-type rats (Fig. 2a), and blocked GABA_A_-mediated inhibition in VTA across phases of the punishment task (Fig. 2d). Post-experiment histology confirmed 13 subjects had bilateral VTA placements (Fig. 2b).

#### Lever-press training and punishment acquisition

Prior to punishment, rats acquired similarly high rates of responding on R1 and R2 across lever-press training (lever: *F*_(1,11)_ = 4.36*, p* = 0.061) (Fig. S9a). Rats then received punishment sessions, where R1 responses were punished with shock (Fig. 2c). Overall, rats were sensitive to this punishment schedule, suppressing responding on punished R1 more than unpunished R2 (*F*_(1,11)_ = 256.75, *p* < 0.001) (Fig. 2e). Rats were also slower to initially press R1 relative to R2 across punishment (*F*_(1,11)_ = 30.79, *p* < 0.001) (Fig. 2f).

To examine the role of GABA inhibition in VTA on this learning, rats received microinfusions of GABA_A_ antagonist bicuculline (A-Bic group; *n* = 6) or control saline (A-Sal group; *n* = 7) into their VTA before the first 2 sessions of punishment. GABA_A_ blockade in VTA, attenuated punishment avoidance during infusion days, such that A-Bic rats suppressed punished R1 responding less than A-Sal rats (*F*_(1,11)_ = 9.05*, p* = 0.012) (Fig. 2e), significantly increasing the number of shocks incurred (*F*_(1,11)_ = 10.60, *p* = 0.008) (Fig. S9d). Bicuculline also attenuated the increase in latency to initially press the punished lever (group*session: *F*_(1,11)_ = 6.58*, p* = 0.026) (Fig. 2f). Crucially, bicuculline had no effect on unpunished R2 response ratios (group: *F*_(1,11)_ = 0.02*, p* = 0.893) or latencies (group: *F*_(1,11)_ = 3.23*, p* = 0.100; group*session: *F*_(1,11)_ = 0.15*, p* = 0.707) during infusion days.

Interestingly, this effect of bicuculline persisted in subsequent non-infusion sessions. Despite 3 additional non-infusion days to learn punishment avoidance, A-Bic rats continued to show less R1 suppression (*F*_(1,11)_ = 6.72*, p* = 0.025; Fig. 2e) and shorter latencies to press R1 (*F*_(1,11)_ = 13.96*, p* = 0.003; Fig. 2f) than A-Sal rats across remaining acquisition sessions. Groups did not differ in R2 suppression (*F*_(1,11)_ = 0.05*, p* = 0.822) or latencies (*F*_(1,11)_ < 0.01, *p* = 0.983) during these sessions. This indicates VTA GABA_A_ blockade during initial punishment produced enduring, consequential insensitivity to punishment.

#### Punishment expression

We then examined the effect of GABA_A_ blockade on expression of learned punishment avoidance. All rats received bicuculline or saline across two punishment sessions (within-subjects, counterbalanced).

The effect of acquisition infusions on punished responding persisted into expression tests; A-Bic rats pressed the punished lever more than A-Sal rats overall (group: *F*_(1,11)_ = 14.00, *p* = 0.003) (Fig. 2g), incurring substantially more shock punishment (*F*_(1,11)_ = 16.15*, p* = 0.002) (Fig. S9e), without any significant group differences in R2 responding (*F*_(1,11)_ = 0.06, *p* = 0.812). There was no acute effect of expression drug on punished responding (*F*_(1,12)_ = 0.29 *, p* = 0.600) (Fig. 2g). However, there was a modest decrease in unpunished responding (*F*_(1,12)_ = 5.60*, p* = 0.036). There was no interaction between acquisition group and expression drug on punished (*F*_(1,11)_ = 0.31*, p* = 0.588) or unpunished (*F*_(1,11)_ = 0.01*, p* = 0.920) response ratios. To further examine whether GABA_A_ blockade during expression test impaired later punishment avoidance, as found for acquisition infusions, we compared punished responding in pre- versus post-bicuculline punishment sessions. Bicuculline had no effect on the subsequent day’s punishment suppression (session: *F*_(1,11)_ = 0.10*, p* = .757; session[A-Sal]: *F*_(1,6)_ = 0.60*, p* = 0.468) (Fig. S9b).

In terms of lever-press latencies, A-Bic rats continued to press R1 faster than A-Sal rats (*F*_(1,11)_ = 5.61*, p* = 0.037) (Fig. 2h), with no acquisition group differences for R2 latencies (*F*_(1,11)_ = 1.20*, p* = 0.297). Expression drug did not significantly affect latencies to press R1 (*F*_(1,11)_ = 0.13*, p* = 0.724) or R2 (*F*_(1,11)_ = 4.68*, p* = 0.053), nor did it interact with acquisition group on lever-press latencies (drug*group: *F*_(1,11)_ = 0.012*, p* = 0.915; drug*group*lever: *F*_(1,11)_ = 0.674*, p* = 0.429).

Taken together, this suggests the effects of VTA GABA blockade on punished behavior are not observed once punishment is already learned. However, there may be a modest role for GABA action in VTA in directing animals towards the unpunished lever.

#### Choice test

Rats were then given two unpunished choice tests (bicuculline vs. saline), each flanked by non-infusion punishment sessions to limit any carry-over effects of these tests (Fig. 2d). Overall, rats preferred the unpunished lever over the punished lever during these tests (*F*_(1,11)_ = 58.16, *p* < 0.001) (Fig. 2i). There was no main effect of acquisition group (*F*_(1,11)_ = 1.072*, p* = 0.323) or choice infusion (*F*_(1,11)_ = 0.531*, p* = 0.481), but there was a significant interaction of group, choice infusion, and lever (*F*_(1,11)_ = 7.586*, p* = 0.019). Consistent with the persistent impairment in punishment avoidance, A-Bic rats pressed the punished lever more than A-Sal rats during saline choice tests (*F*_(1,11)_ = 15.18*, p* = 0.002). This was not observed during bicuculline choice tests (*F*_(1,11)_ = 0.237*, p* = 0.636) tests; bicuculline significantly increased selection of R1 in A-Sal (*F*_(1,6)_ = 9.39*, p* = 0.022) but not A-Bic (*F*_(1,5)_ = 2.73*, p* = 0.159) rats. Acquisition group did not interact with choice infusion for unpunished responding (*F*_(1,11)_ = 1.57*, p* = 0.236).

#### Effects of VTA disinhibition on open field activity

Rats then received bicuculline or saline infusions (within-subjects, counterbalanced) prior to an open field test. A-Bic rats were hyperactive compared to A-Sal rats (*F*_(1,11)_ = 20.18*, p* = 0.001) (Fig. 2j).There was no acute effect of bicuculline (*F*_(1,11)_ = 0.01*, p* = 0.972), nor any interaction between acquisition group and open field infusion (*F*_(1,11)_ = 2.02*, p* = 0.183), on distance traveled.

In summary, the findings of Experiment 2 indicate that preventing GABA_A_ inhibition in VTA during initial punishment learning, but not already-learned punishment, drives an enduring impairment in punishment avoidance and hyperactivity.

### Experiment 3: Effects of chemogenetic activation of VTA_DA_ neurons during punishment

The findings of Experiment 2 broadly conform with the notion that inhibition of midbrain dopamine neurons mediates aversive learning. However, GABA also acts on non-dopamine neurons within VTA including GABA interneurons that inhibit VTA_DA_ [16], so the effects of bicuculline into VTA could be mediated by effects on other VTA populations. We therefore tested whether direct upregulation of VTA_DA_ neuron activity during punishment learning also produces enduring impairments in punishment avoidance.

**Fig. 3:**
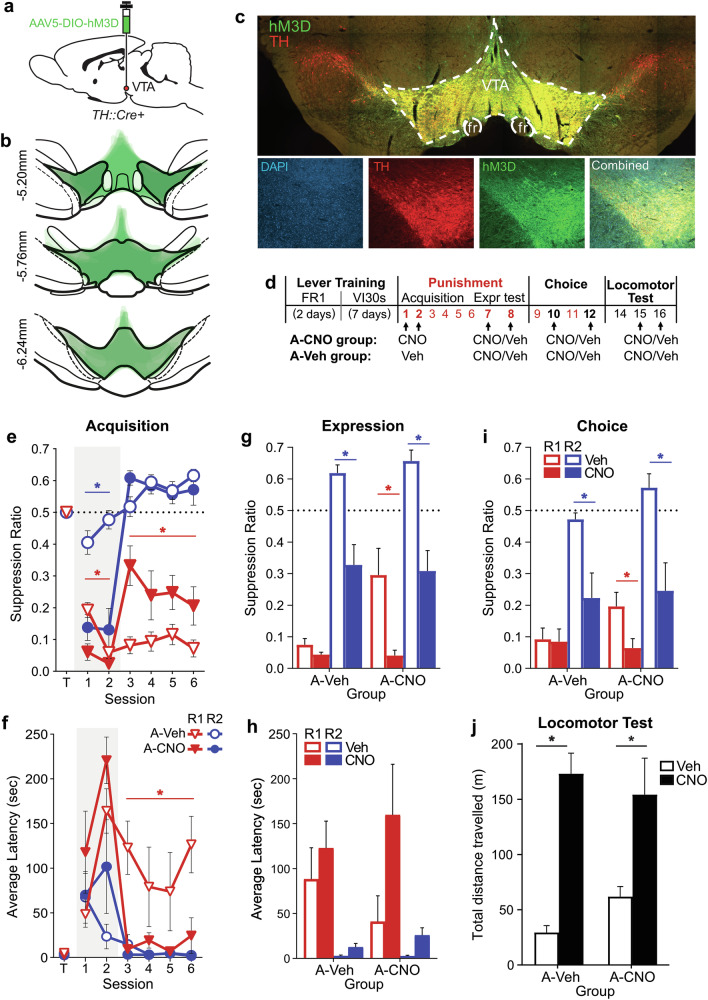
Effects of chemogenetic activation of VTA_DA_ neurons during punishment. **a** TH::Cre+ rats received Cre-dependent excitatory hM3D DREADD bilaterally into the VTA. **b** hM3D expression across animals included in analyses (*N* = 10). **c** Example expression of hM3D within VTA. **d** Timeline of task sessions, with arrows indicating when subjects received i.p. injections of CNO and/or vehicle control (Veh). A-CNO vs. A-Veh groups received CNO vs. Veh (respectively) before the first 2 sessions of punishment. All groups received CNO vs. Veh (within-subjects, order counterbalanced) in subsequent punishment expression, choice, and locomotor tests. **e** Mean ± SEM lever suppression ratios by acquisition group across last day of training (T) and punishment acquisition sessions. Grey shaded area indicates injection sessions. CNO acutely (but incompletely) reduced responding. On subsequent non-injection days, A-CNO rats exhibited a persistent deficit in punishment avoidance. **f** Mean ± SEM lever-press latencies by acquisition group across last day of training (T) and punishment acquisition sessions. CNO administration did not significantly affect lever-press latencies acutely. However, A-CNO rats pressed the punished R1 lever significantly faster than A-Veh rats on subsequent non-injection days. **g** Mean ± SEM lever suppression ratios during punishment expression tests. CNO injections acutely (but incompletely) reduced responding across acquisition groups. A-CNO rats continued to press punished R1 more than A-Veh rats during control injections. **h** Mean ± SEM lever-press latencies during punishment expression tests. **i** Mean ± SEM suppression ratios during choice tests. **j** Mean ± SEM distance traveled during locomotor tests. CNO acutely increased distance traveled across groups. *p < 0.05.

To examine this, we expressed excitatory designer receptor hM3D in VTA_DA_ neurons of male TH::Cre+ rats (Fig. 3a, b). Rats then underwent the same task described for Experiment 2, except rats received systemic injections of CNO or control vehicle instead of microinfusions; A-CNO group (*n* = 5) received CNO during acquisition injections whereas A-Veh group (*n* = 5) received vehicle. If effects of GABA blockade are mediated by disinhibition of VTA_DA_, chemogenetic excitation of VTA_DA_ should reproduce the effects of Experiment 2.

#### Lever-press training and punishment acquisition

Prior to punishment, rats acquired similar rates of pressing on both levers (lever: *F*_(1,8)_ = 1.57, *p* = 0.246; lever*group: *F*_(1,8)_ = 0.08, *p* = 0.790) (Fig. S10a). During punishment, R1 responding was suppressed (ratio: *F*_(1,8)_ = 138.39, *p* < 0.001; latencies: *F*_(1,8)_ = 14.22, *p* = 0.005), relative to unpunished R2 responses (Fig. 3e, f).

Chemogenetic activation of dopamine neurons during initial punishment sessions (A-CNO group) produced acute suppression of both punished (group: *F*_(1,8)_ = 10.69, *p* = 0.011) and unpunished responding (group: *F*_(1,8)_ = 28.91, *p* = 0.001) (Fig. 3e). It is worth noting all A-CNO animals still made responses on both levers, with a non-significant trend towards more unpunished responding (lever [A-CNO only]: *F*_(1,4)_ = 5.95, *p* = 0.071). All animals pressed enough to receive shock(s) during initial punishment (Fig. S10d).

On following non-injection days, responding rebounded in A-CNO animals. A-CNO group pressed the unpunished lever at similarly high rates to A-Veh group (*F*_(1,8)_ = 0.03, *p* = 0.876). However, A-CNO group suppressed punished responding significantly less (*F*_(1,8)_ = 7.08, *p* = 0.029), engaged the punished lever significantly faster (*F*_(1,8)_ = 5.85, *p* = 0.042), and received many more shocks (*F*_(1,8)_ = 10.33, *p* = 0.012) (Fig. S10d) than A-Veh animals across non-injection days. This was not solely attributable to delayed learning due to initially reduced responding as A-CNO R1 responding (and shocks incurred) across these sessions remained higher than even the first session of punishment for A-Veh group. This indicates that activation of VTA_DA_ during initial punishment learning produces enduring punishment insensitivity, as found for VTA disinhibition using GABA_A_ blockade.

#### Punishment expression

Prior to days 7 and 8 of punishment, rats received CNO or vehicle injections (within-subjects, counterbalanced). A-CNO animals continued to show punishment insensitivity; they pressed the punished (*F*_(1,8)_ = 6.46, *p* = 0.035) but not unpunished (*F*_(1,8)_ = 0.78, *p* = 0.403) lever significantly more than A-Veh following vehicle injections (Fig. 3g), As observed during acquisition injections, CNO administration acutely suppressed responding (drug: *F*_(1,8)_ = 39.99, *p* < 0.001) (Fig. 3g) and increased latencies to press levers (drug: *F*_(1,8)_ = 6.56, *p* = 0.034) (Fig. 3h), without eliminating discriminated responding (lever[CNO]: *F*_(1,8)_ = 47.38, *p* < 0.001).

#### Choice test

Rats were then given two choice tests (CNO vs. Veh, counterbalanced). Overall, rats preferred the unpunished over punished lever (*F*_(1,8)_ = 78.95, *p* < 0.001) and CNO broadly suppressed responding (drug: *F*_(1,8)_ = 23.52, *p* = 0.001) (Fig. 3i). Acquisition group did not significantly interact with effects of lever or choice injection (all *F*_(1,8)_ ≤ 1.881, *p* ≥ 0.207).

#### Open field activity

A hardware failure resulted in no data being collected for an A-CNO animal during their Veh test; this animal was thus excluded from analyses. VTA_DA_ activation via CNO profoundly increased distance traveled in the open field test (drug: *F*_(1,7)_ = 44.60, *p* < 0.001) (Fig. 3j). Locomotor activity did not significantly depend on acquisition group (group: *F*_(1,7)_ = 0.74, *p* = 0.741; group*drug: *F*_(1,7)_ = 2.122, *p* = 0.189). However, it is worth noting a trend towards increased locomotor activity in A-CNO group relative to A-Sal during the control Veh test (*F*_(1,7)_ = 9.112; *p* = 0.019), mirroring the group difference observed in Experiment 2.

## Discussion

Avoiding punishment is a core component of adaptive behavior. The current study explored the role of VTA_DA_ inhibition in punishment learning and choice. Using fiber photometry to record VTA_DA_ dynamics (Experiment 1), we observed phasic increases in VTA_DA_ neuron activity and GABA input around response-elicited appetitive and aversive events. VTA_DA_ activity was more reward-biased, whereas GABA input was punisher-biased (at least during initial punishment). This generally conforms with traditional theories that GABA inhibition of VTA_DA_ during unpredicted adverse events drives punishment learning [4, 5]. Testing this, we blocked GABA_A_ inhibition in VTA (Experiment 2) or directly activated VTA_DA_ neurons (Experiment 3) and showed disinhibiting VTA during initial punishment learning induced long-term impairments in punishment avoidance. This accords with previous studies that show chemogenetic activation of VTA_DA_ promotes risky decision-making [31, 32]. Interestingly, we found acute disinhibition of VTA after punishment was learned did not induce subsequent insensitivity. Together, these findings suggest long-term avoidance depends upon a critical window of GABA-mediated VTA_DA_ inhibition during initial punishment learning.

### Roles for VTA inhibition in punishment learning and choice

One explanation for the enduring, time-sensitive effect of GABA_A_ blockade and hM3D activation on avoidance is that these manipulations prevented normal inhibitory prediction error signaling within VTA_DA_ during the initially unexpected shock outcomes during early punishment sessions. In theory, this would undermine aversive learning about the antecedent action. Indeed, we found punisher-elicited GABA efflux onto VTA_DA_ neurons was most pronounced during initial punishment, as predicted by aversive prediction error accounts of VTA_DA_. Although congruent, this interpretation is speculative, as manipulations in this study were not restricted to the moment of shock delivery. However, previous studies have shown brief optogenetic inhibition of VTA_DA_, delivered in the same manner as shocks were in the current study, was sufficient to drive punishment avoidance [13]. Together, these findings suggest punisher-elicited inhibition of VTA_DA_ is both sufficient and necessary for the acquisition of punishment avoidance.

It also possible that manipulations increased punished responding indirectly via effects on reward processing. Although VTA manipulations did not cause increased unpunished reward-seeking, the possibility of ceiling effects on unpunished responding is a critical consideration. Previous studies using the same task parameters have shown neural manipulations can increase unpunished reward-seeking [21, 33], suggesting unpunished responding is not typically at ceiling in this task. Thus, the absence of any trend towards increased unpunished reward-seeking in the current study suggest increased punished responding is not attributable to increased reward motivation. Indeed, the only significant effects of VTA disinhibition on unpunished behavior were acute reductions in unpunished response rates and slower latencies to make unpunished responses. Therefore, increased punished responding following VTA disinhibition seems attributable to perturbed punishment-related over reward-related processing within VTA.

One observation that deviates from this punishment-driven VTA_DA_ inhibition account was that VTA_DA_ population activity generally increased during the footshock punisher, despite concurrent increases in GABA input. This highlights the dissociation between VTA_DA_ activity and its inhibitory inputs. A key consideration here is the heterogeneity of signaling across VTA_DA_ neuron subtypes. Seminal reports of VTA_DA_ being broadly inhibited by aversive events were from neurons with a specific electrophysiological signature, which ignored VTA_DA_ neuron subtypes that do not share this signature and are excited by aversive events [34, 35]. Measurement from the broader population of genetically-defined VTA_DA_ neurons, as done here, often report excitatory VTA_DA_ transients to aversive events [5, 30, 34, 36]. The current study does not provide insight into whether manipulation effects were mediated by specific VTA_DA_ subtypes or circuits [34, 35, 37]. It is plausible that manipulation effects were specifically due to actions on subpopulations that receive increased GABA input during punishers, but further exploration of the cell-type and circuit basis of effects are needed. Indeed, VTA_DA_ neurons project to several regions strongly implicated in punishment avoidance, such as nucleus accumbens (NAc) and basolateral amygdala [4, 16, 34, 38]. Elevated dopamine in nucleus accumbens is associated with increased risk-taking under punishment [32, 39], suggesting disinhibition within the VTA-NAc circuit could mediate the effects observed in the current study.

Another finding was that GABA_A_ blockade acutely increased punished lever-pressing during choice test for acquisition control (A-Sal) rats, suggesting an additional role for GABA input to VTA in sculpting behavior when faced with a discrete choice. This effect of VTA disinhibition on choice was not observed in the hM3D experiment. One explanation for this discrepancy between experiments was that hM3D activation of VTA_DA_ acutely suppressed lever-pressing, which might have undermined detection of a corresponding increase in punished responses during choice test. Indeed, CNO administration during choice test significantly suppressed all lever-pressing except punished responses in A-Veh rats. This lack of effect on punished responding could represent a floor effect, but could also represent counteracting effects of VTA_DA_ activation on punished choice. Alternatively, this discrepancy between VTA GABA_A_ blockade and direct VTA_DA_ activation during choice test could represent a dissociation in how VTA GABA input and VTA_DA_ neuron activity sculpt behavior. Indeed, acute suppression of instrumental responding and increased locomotion seen with VTA_DA_ activation was not observed following VTA GABA_A_ blockade suggesting these manipulations are not neurally equivalent. Given GABA input is only part of the milieu dictating VTA_DA_ activity [16], further research is needed to explore how various excitatory and inhibitory inputs to VTA_DA_, and resultant VTA_DA_ activity, contribute to punishment learning and behavior.

### Considerations on external validity and broader implications

A limitation of the current study is that neural data predominantly derives from male subjects, preventing examination of potential sex differences. In general, females tend to be more sensitive to punishment than males [41, 45–48]. Although our supplemental findings indicate minimal sex differences in punishment avoidance in the task used here, a lack of behavioral differences do not preclude dissociated neural underpinnings [49]. Indeed, there are known sex differences in dopamine release and dopamine receptor expression [50, 51], including evidence for sex differences in dopaminergic contributions to punishment avoidance [52, 53]. Further research into whether findings of the current study depend on sex are needed.

Another key question is whether the enduring insensitivity induced by VTA_DA_ disinhibition represents a broad behavioral deficit that would carry over to new punishing scenarios, or is instead specific to the punished action, punisher, and/or context in which VTA_DA_ disinhibition occurred. For example, VTA disinhibitions may have specifically altered the motivational value of the experienced shock (e.g., via counterconditioning [40]). Alternatively, VTA disinhibitions may have undermined normal Action-Punisher association learning (a common locus for naturally-occurring punishment insensitivity [41, 42]). This latter idea accords with newer theories of dopamine which argue dopamine signals do not simply compute model-free prediction errors, but instead help build cognitive maps of relationships between actions, cues and outcomes [43, 44]. Our observation that disinhibition-induced insensitivity was accompanied by locomotor hyperactivity in a different context suggests the perturbation extends beyond the punishment scenario in which VTA signaling was disrupted, but it will be important to examine whether insensitivity is observed with other actions, punishers, or contexts. This raises a related question of the causal relationship between effects on punishment and locomotion. Hyperactivity itself does not necessarily drive punished responding, as demonstrated by VTA_DA_ activations acutely increasing locomotion but suppressing responding. Nevertheless, it is plausible that effects on punishment and locomotion are functionally related. We did not measure locomotion during punishment learning; future research could examine this to explore the connection between these dual effects of VTA disinhibition.

More broadly, the finding that brief perturbations of dopamine can cause long-lasting impairments in harm avoidance has special relevance for substance addictions, which are diagnostically characterized by the persistence of drug-seeking and -taking despite negative consequences [54]. Addictive substances across drug classes are known to artificially elevate dopamine and/or disrupt inhibitory input to dopamine neurons [55]. The current study highlights a potential mechanistic connection between these substances and their tendency to drive compulsive (i.e., punishment insensitive) drug-taking. Substance-induced deficits in appropriately learning about the negative consequences of drug-seeking may coalesce with addictive substances’ other effects on cognition, motivation, and neural circuit functioning [56–61] to drive the complex and difficult-to-treat nature of drug addiction. A key question that follows is whether deficits in harm avoidance observed here can be reversed or, in the case of anticipated hyperdopaminergic states (e.g., pharmacotherapies [62]), ameliorated.

## Conclusions

In summary, our findings build upon existing work investigating VTA dopamine and GABA in reward and aversion [63–65], and identify inhibitory input to VTA_DA_ as a critical mechanism for adaptive punishment avoidance. Disrupting inhibition within VTA or directly upregulating VTA_DA_ activity during initial punishment learning caused long-term deficits in avoidance. Further investigation is needed to identify the psychological nature of these deficits, the specific circuits and plasticity mechanisms mediating these effects, and how they might be reversed to restore adaptive choice.

## Supplementary information

Supplemental Material

## Data Availability

Data associated with the current study are available from the corresponding author on request.
