# Supplementary material for: Disinhibition of ventral tegmental area during initial punishment learning causes enduring punishment insensitivity
Source: Neuropsychopharmacology. 2026 Feb 17;51(6):1045–55. doi: 10.1038/s41386-026-02368-4 (PMC13125270; doi:10.1038/s41386-026-02368-4)
Supplement: Supplementary file 1 — Supplemental Material [file 41386_2026_2368_MOESM1_ESM.docx]

**SUPPLEMENTAL MATERIAL**

**Materials and Methods**

**Behavior Apparatus**

Across experiments, operant behavioral testing was conducted in identical experimental chambers (24 [length] x 30 [width] x 21cm [height]; Med Associates Inc., VT, USA). Each chamber was enclosed in sound- and light-attenuating cabinets (40 × 56 × 56 cm) and fitted with fans for ventilation and background noise. Chambers were made of Perspex rear-wall, ceiling and hinged front-wall, and stainless-steel sidewalls. Chamber floors were made of stainless-steel rods (4mm in diameter) spaced 15mm apart. A recessed magazine (3cm in diameter) within a 4cm x 4cm hollow in the right-side chamber wall received grain pellets (45mg; Bio-Serv) from an external automatic hopper. Infrared photocells detected entries into the magazine. Infrared digital cameras, attached to the ceiling of the cabinets, recorded all activity within the chamber from above. For fiber photometry experiments, a patch cable was threaded into the chamber through a ceiling hole and supported by a counterweighted gimbal holder.

Locomotor tests were conducted in open field chambers (43.2 × 43.2 × 30.5 cm; Med Associates) that tracked movement via 16-beam infrared arrays located along X- and Y-axes.

**Fiber photometry apparatus**

Two Doric LEDs, controlled via dual channel LED drivers, provided 465 nm (experimental signal) and 405 nm (isosbestic control signal) excitation light. Resulting fluorescence (~525nm) was measured using femtowatt photoreceivers (Newport 2151). Doric Dual Fluorescence Mini Cube (FMC2, Doric Lenses) relayed excitation/fluorescence wavelengths to/from pre-bleached patch cable and fiber optic implant. A real-time processor (RZ5P, Tucker Davis Technologies) controlled and modulated excitation lights (465 nm: 209 Hz; 405 nm: 331 Hz), as well as demodulated and low-pass filtered (3 Hz) fluorescence signals. The RZ5P also received Med-PC signals to record behavioral events in real-time. Light intensity at the tip of the patch cable was maintained at 10-40 µW across sessions.

Multimode fiber optic cannulae implants and patch cables (0.39 NA, 400μm core) were constructed using materials from Thor Labs (Newton, NJ, USA).

**Surgery**

For neural manipulation experiments, rats were anaesthetized using 1.3 ml/kg ketamine (100 mg/ml; Ketapex; Apex Laboratories, Sydney, Australia) and 0.2 ml/kg muscle relaxant, xylazine (20 mg/ml; Rompun; Bayer, Sydney, Australia) (i.p.). For fiber photometry experiments, rats were anesthetized with isoflurane (5% induction; 2% maintenance).

Following anesthetic induction, rats were placed in a stereotaxic frame (Model 900, Kopf, Tujunga, CA, USA), with the incisor bar maintained at approximately 3.3 mm below horizontal to achieve a flat skull position.

Immediately following surgery, animals were given i.p. injections of antibiotics. For neural manipulation experiments, 0.3ml procaine penicillin solution (300mg/ml Benicillin; Illium) and 0.3ml cefazolin (100mg/ml) was given. For fibre photometry experiments, Duoplicin (0.15ml/kg) was given instead.

**Perfusions and immunohistochemistry**

For Experiments 1 and 3, rats were anesthetized with i.p. injections of sodium pentobarbital (100mg/kg) and perfused with 0.9% saline solution containing 1% sodium nitrate and heparin (5000 IU/ml), followed by 4% paraformaldehyde in phosphate buffer solution (PB; 0.1M). Brains were extracted, incubated in 20% sucrose solution for cryoprotection, sliced coronally (40μm) through VTA using a cryostat and stored in PB solution with 0.1% sodium azide at 4°C.

Brain tissue was washed in PB, incubated in PBT-X solution (10% horse serum [NHS], 0.5% Triton X-100 in PB) for 2 hours, and then incubated in PBT-X solution (2% NHS, 0.2% TritonX-100 in PB) with primary antibody (Experiment 1: 1:1000 rabbit anti-GFP [ThermoFisher Scientific, #A11122], 1:1000 sheep anti-TH [Sigma Aldrich, #AB1542]; Experiment 3: 1:1000 rabbit anti-mCherry [Abcam, #ab167453], 1:2000 sheep anti-TH [Sigma Aldrich, #AB1542]) and at room temperature for 24hr. Tissue was then washed with PB and incubated overnight in PBT-X (2% NHS, 0.2% TritonX-100 in PB) with secondary antibody (Experiment 1: 1:1000 AlexaFluor 488 anti-rabbit [ThermoFisher Scientific, #A21206], 1:1000 Cy3 donkey anti-sheep [Jackson ImmunoResearch, #713-095-147]; Experiment 3: 1:500 AlexaFluor 488 donkey anti-rabbit [Invitrogen, #A21206], 1:1000 AlexaFluor 555 donkey anti-sheep [Invitrogen, #A21436]; 1:1000 AlexaFluor 594 donkey anti-goat [Invitrogen, #A11058]). Tissue was washed with PB and mounted onto gelatinized slides. Slides were left to dry and then cover-slipped. Biosensor expression and cannula placements were verified using fluorescent microscopy. Animals were excluded from analyses if fiber tip and biosensor expression could not be confirmed as co-localized in VTA.

For pharmacology (Experiment 2), rats were anesthetized with i.p. injections of sodium pentobarbital (100mg/kg). Unfixed brains were extracted, frozen and sliced coronally (40 µm) through VTA using a cryostat. Each section was collected and subsequently stained with cresyl violet for histological examination. Animals were excluded from analyses if guide cannulae were not bilaterally located within VTA.

**Sex differences in punishment task behavior**

To assess the influence of sex on behavior in the task used across this study, 16 male and 16 female experimentally-naïve rats were run through the punishment task. Animals in this experiment were not subjected to any surgical procedures or neural manipulation; this was a purely behavioral study examining the biological variable of sex.

Behavioral procedures were equivalent to those described in the main text. Briefly, rats were first trained to press 2 levers for food. In the first 2 sessions (1hr each), levers were presented concurrently and every press was rewarded; each lever retracted after 25 presses. In the next 7 sessions (40min each), levers were individually presented (5min alternating blocks) and presses were rewarded on VI30s schedule. Subsequent punishment sessions were identical to VI30s training sessions, except every 10^th^ press on the designated punished lever resulted in immediate 0.5sec 0.5mA footshock. After 5 sessions of punishment, animals received a choice test (10min) in which both levers were presented concurrently and no shocks were delivered.

The key question was whether males differed from females, behaviorally, during punishment and choice sessions. Animals were excluded if they showed a spurious preference for one lever by the end of lever training (one lever comprising more than two-thirds of all lever-presses in final VI30s session, despite matched reward and solo lever presentations); 2 females were excluded based on this criterion. Findings are reported in **Figure S1**. In brief, no sex differences in punished responding were observed. Males tended to make more unpunished responses than females across pre-punishment, punishment, and choice test, but no differences were observed once accounting for baseline differences in response rates (**Figure S1b**).

**Relationships between event-related photometry signals and behavior**

Temporally-defined relationships between event-related VTA dynamics and behavior per session were examined via Pearson correlations of response bias x outcome- or action-related signal bias (**Figure S5**).

Response bias per subject was calculated via a R1:R2 preference ratio, as per previous studies^13^: R1 rate/(R1 rate + R2 rate). This provides a self-normalized measure of response preference, with lower scores indicating less preference for R1 relative to R2. Outcome-related signal bias per subject was calculated as the difference between Shock and Pellet activity (Shock–Pellet difference waveform), which provides a self-normalized measure of signal around aversive vs. appetitive outcomes. Action-related signal bias per subject was calculated as the difference between R1 and R2 activity (R1–R2 difference waveform), which provides a self-normalized measure of signal around punished R1 vs. unpunished R2 actions. These measures were chosen (instead of using each event signal [i.e., Pellet, Shock, R1, R2] and behavior measure [e.g., R1 lever suppression, R2 lever suppression, R1 press rates, R2 press rates]) to maximize the relevance and consistency of data used in these analyses while constraining the overall number of analyses.

Pearson *r* and corresponding *p* values for preference ratio against event signal across the event window was determined used MATLAB’s inbuilt *corr* function. As per peri-event bootstrapped confidence interval analyses, a temporal threshold of 1/3sec (low-pass filter window) was used to limit Type 1 error.

**Isolated peri-event activity via General Linear Modelling**

To address potential contamination of peri-event signals by other nearby event signals (e.g., signals from nearby outcomes or other lever-presses contaminating average lever-press-alone signal), we employed a general linear modelling approach to remove estimated contributions of other events to peri-event activity. This modelling approach was conducted using custom MATLAB scripts made available at <https://github.com/philjrdb/FibPhotom>.

First, normalized session dF/F for a given task phase (Training, Pun 1, Late Pun, or Choice) were concatenated across subjects. Timestamps for task event onsets were used to build a linear model of event-related session activity. This was done by estimating dF/F change attributable to each event type (R1 action, R2 action, pellet delivery, footshock, magazine entry after pellet delivery, other magazine entry, R1 lever extension, R2 lever extension) across a peri-event window (-3sec to +7sec around event onset). Behavioral events (actions, magazine entries) used the full peri-event window, whereas environmental events (outcomes, lever extensions) excluded the pre-event portion of the window to avoid overfitting. Initial transient estimates were the mean peri-event signal per event type; these estimates were iteratively adjusted according to model residuals around each event type. Specifically, a prediction of session dF/F was created by adding relevant transient estimates to event timestamps. Average peri-event residuals (predicted – actual dF/F), which represent biased predictions due to summation of overlapping event signals, were used to concurrently adjust each event’s peri-event estimate, such that timepoints with positive vs. negative residuals (over- vs. under-prediction) were proportionally decreased or increased, respectively. These new peri-event estimates were then used to build another prediction of session dF/F. This process was repeated across 2000 iterations, driving transient estimates towards an equilibrium where there is no bias in peri-event residual. Final transient estimates obtained from this process represent isolated activity patterns attributable to each event. Across iterations, overall residuals (the difference between predicted and actual dF/F) exponentially decreased to an asymptote, indicating that peri-event estimate-based modelling of dF/F had become more accurate.

Transient estimates were then used in a General Linear Model (GLM), where each trial’s peri-event estimates were multiplied by a coefficient to account for magnitude differences in event-related activity across trials. Optimal coefficients were solved using the MATLAB pseudo-inverse (*pinv*) function. To obtain model-isolated event activity, dF/F signals around event trials were collated after subtracting GLM-predicted contributions of all other events (including predicted contributions of other trials of the same event type). These isolated peri-event signals were analyzed using bootstrapped confidence intervals in the same manner as conventional peri-event analyses (as described in main Methods). Isolated peri-event signals are reported in **Figure S7**.


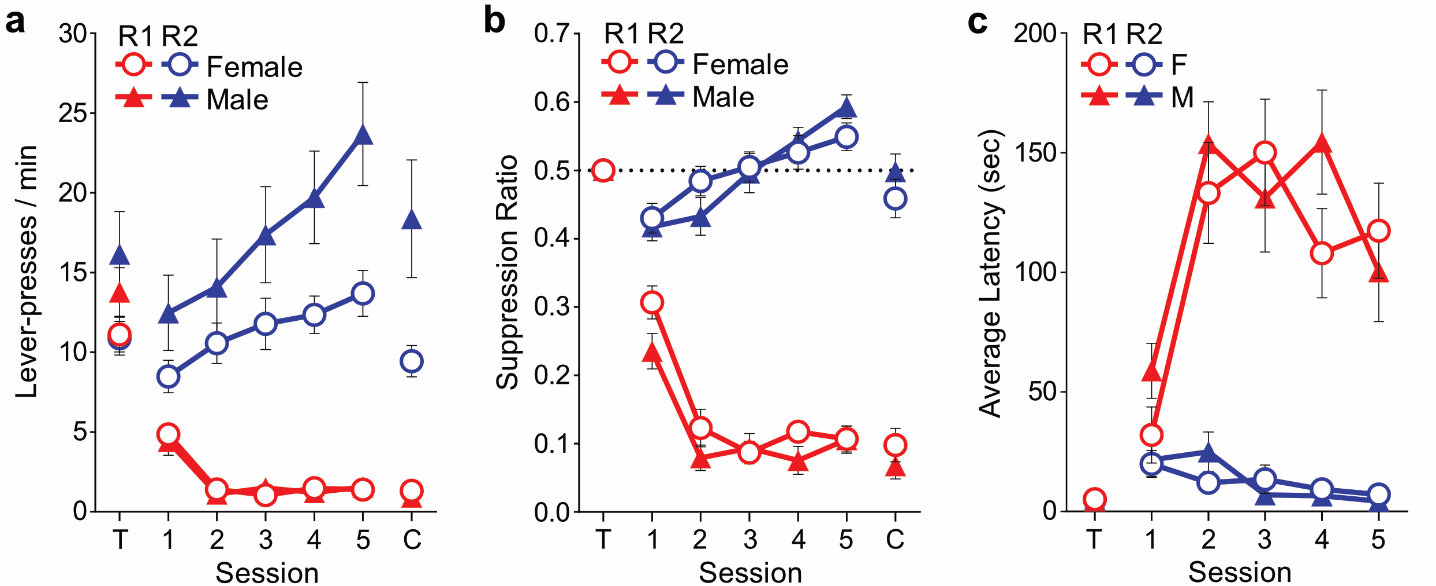


**Figure S1. Sex differences in punishment task behavior. [A]** Mean ± SEM lever-press rates for R1 and R2 across the last day of training (T), punishment (1-5), and choice (C) sessions for female (*n*=14) and male (*n*=16) rats. There was no significant preference between levers before punishment (lever: *F*_(1,28)_=1.607, *p*=.215; lever*sex: *F*_(1,28)_=2.437, *p*=.130). There was a trend towards greater overall responding in males before punishment but this did not reach statistical significance (*F*_(1,28)_=2.753, *p*=.108). During punishment, all subjects exhibited a strong preference for the unpunished R2 lever during punishment (lever: *F*_(1,28)_=59.30, *p*<.001), with a trend towards greater discrimination in males (lever*sex: *F*_(1,28)_=3.657, *p*=.066). This discrimination persisted into choice tests (lever: *F*_(1,28)_=37.51, *p*<.001), with a significant lever x sex interaction (*F*_(1,28)_=4.986, *p*=.034). This interaction was driven by males pressing the unpunished lever more than females (sex[R2]: *F*_(1,28)_=4.847, *p*=.036); no differences in punished responding was observed (sex[R1]: *F*_(1,28)_=0.656, *p*=.425). **[B]** Mean ± SEM lever suppression ratios per lever across sessions by sex. When normalized by pre-punishment rates of responding, no sex differences in responding were observed across punishment sessions (lever: *F*_(1,28)_=356.8, *p*<.001; lever*sex: *F*_(1,28)_=0.478, *p*=.471; sex: *F*_(1,28)_=0.929, *p*=.343) and choice test (lever: *F*_(1,28)_=182.4, *p*<.001; lever*sex: *F*_(1,28)_=1.405, *p*=.246; sex: *F*_(1,28)_=0.059, *p*=.809). **[C]** Mean ± SEM average lever-press latencies per lever across sessions by sex. No differences in latencies were observed prior to punishment (lever: *F*_(1,28)_=0.174, *p*=.680; lever*sex: *F*_(1,28)_=0.357, *p*=.555; sex: *F*_(1,28)_=0.058, *p*=.811). During punishment, animals were significantly slower to respond on the punished relative to unpunished lever (lever: *F*_(1,28)_=100.2, *p*<.001); this did not depend on sex (lever*sex: *F*_(1,28)_=0.298, *p*=.589; sex: *F*_(1,28)_=0.365, *p*=.551).


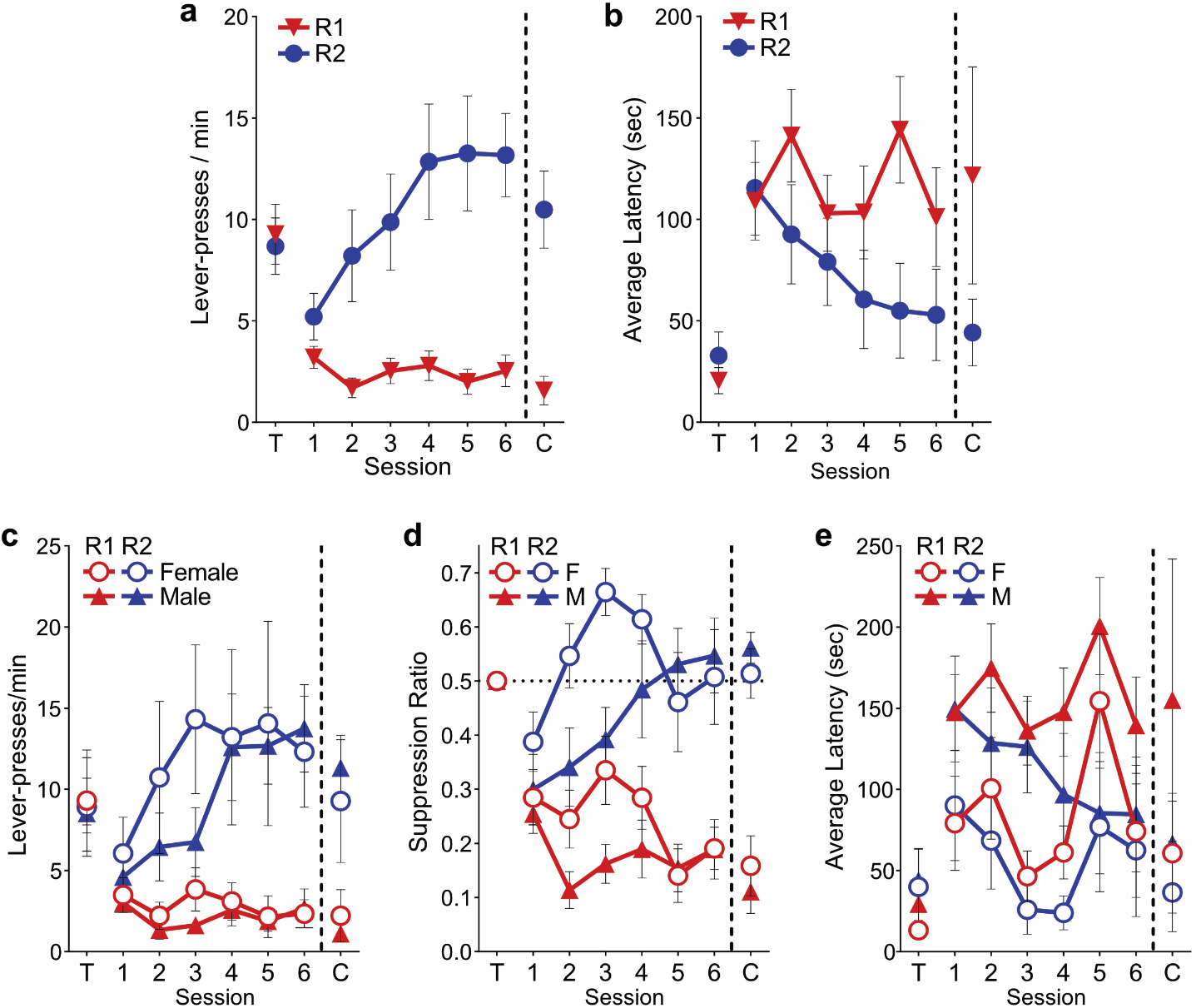


**Figure S2. Supplementary behavioral results for Experiment 1. [A]** Mean ± SEM lever-press rates for R1 and R2 across the last day of training (T), punishment (1-6), and choice (C) sessions (*N* = 14). All subjects exhibited a strong preference for the unpunished lever R2 during punishment and choice tests. **[B]** Mean ± SEM average lever-press latencies per lever across sessions. No differences in latencies were observed prior to punishment (lever: *F*_(1,16)_=1.86, *p*=.192). During punishment, animals were significantly slower to respond on the punished relative to unpunished lever (lever: *F*_(1,16)_=10.74, *p*=.005). **[C]** Mean ± SEM lever-press rates across sessions by sex (female: *n* = 7; male: *n* = 10). There were no significant sex differences in lever-press rates for pre-punishment (sex: *F*_(1,15)_=0.006, *p*=.938; lever*sex: *F*_(1,15)_=0.044, *p*=.837), punishment (sex: *F*_(1,15)_=0.387, *p*=.543; lever*sex: *F*_(1,15)_=0.162, *p*=.693), or choice (sex: *F*_(1,15)_=0.053, *p*=.821; lever*sex: *F*_(1,15)_=0.514, *p*=.484). **[D]** Mean ± SEM lever suppression ratios across sessions by sex. There were no significant sex differences for punishment (sex: *F*_(1,15)_=2.30, *p*=.150; lever*sex: *F*_(1,15)_=0.144, *p*=.709; lever*session*sex: *F*_(1,15)_=1.22, *p*=.286), or choice (sex: *F*_(1,15)_<0.01, *p*=.999; lever*sex: *F*_(1,15)_=1.20, *p*=.290). **[E]** Mean ± SEM average lever-press latencies per lever across sessions by sex. There were no significant sex differences for pre-punishment (sex: *F*_(1,15)_<0.01, *p*=.999; lever*sex: *F*_(1,15)_=1.90, *p*=.188), punishment (sex: *F*_(1,15)_=1.49, *p*=.241; lever*sex: *F*_(1,15)_=0.745, *p*=.402), or choice (sex: *F*_(1,15)_=0.975, *p*=.339; lever*sex: *F*_(1,15)_=0.671, *p*=.426).

**
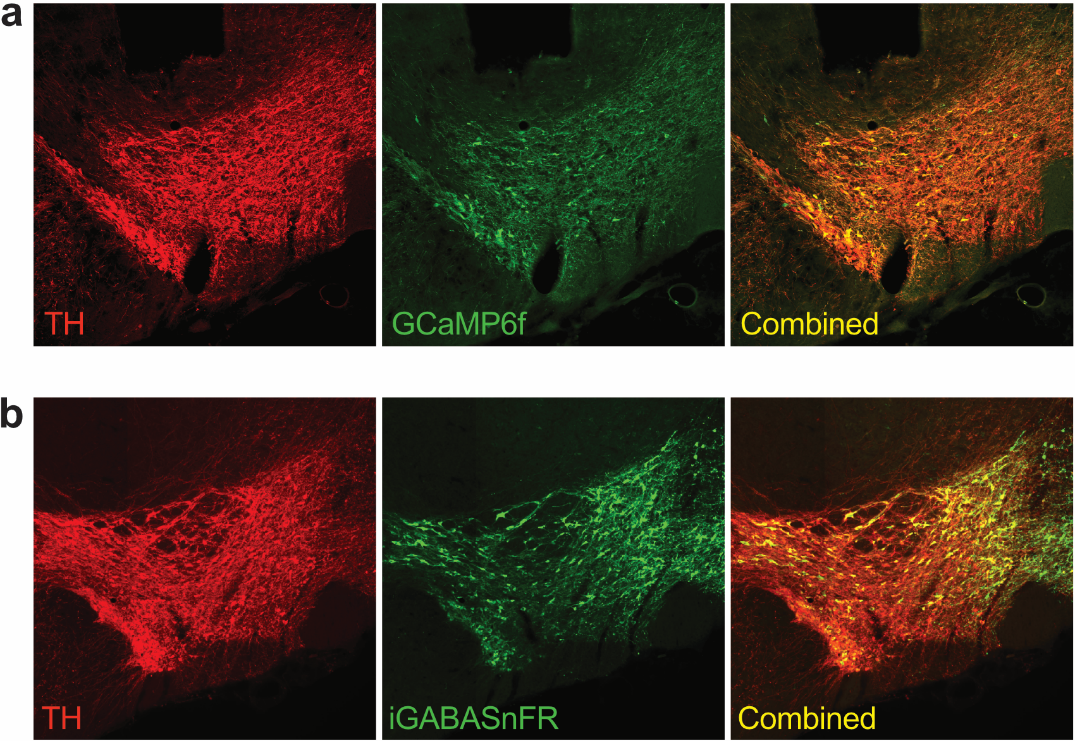
**

**Figure S3. Verification of Cre-dependent sensor expression. [A]** Example expression of GCaMP6f in VTA tyrosine hydroxylase (TH) neurons. **[B]** Example expression of iGABASnFR in VTA TH neurons.

**
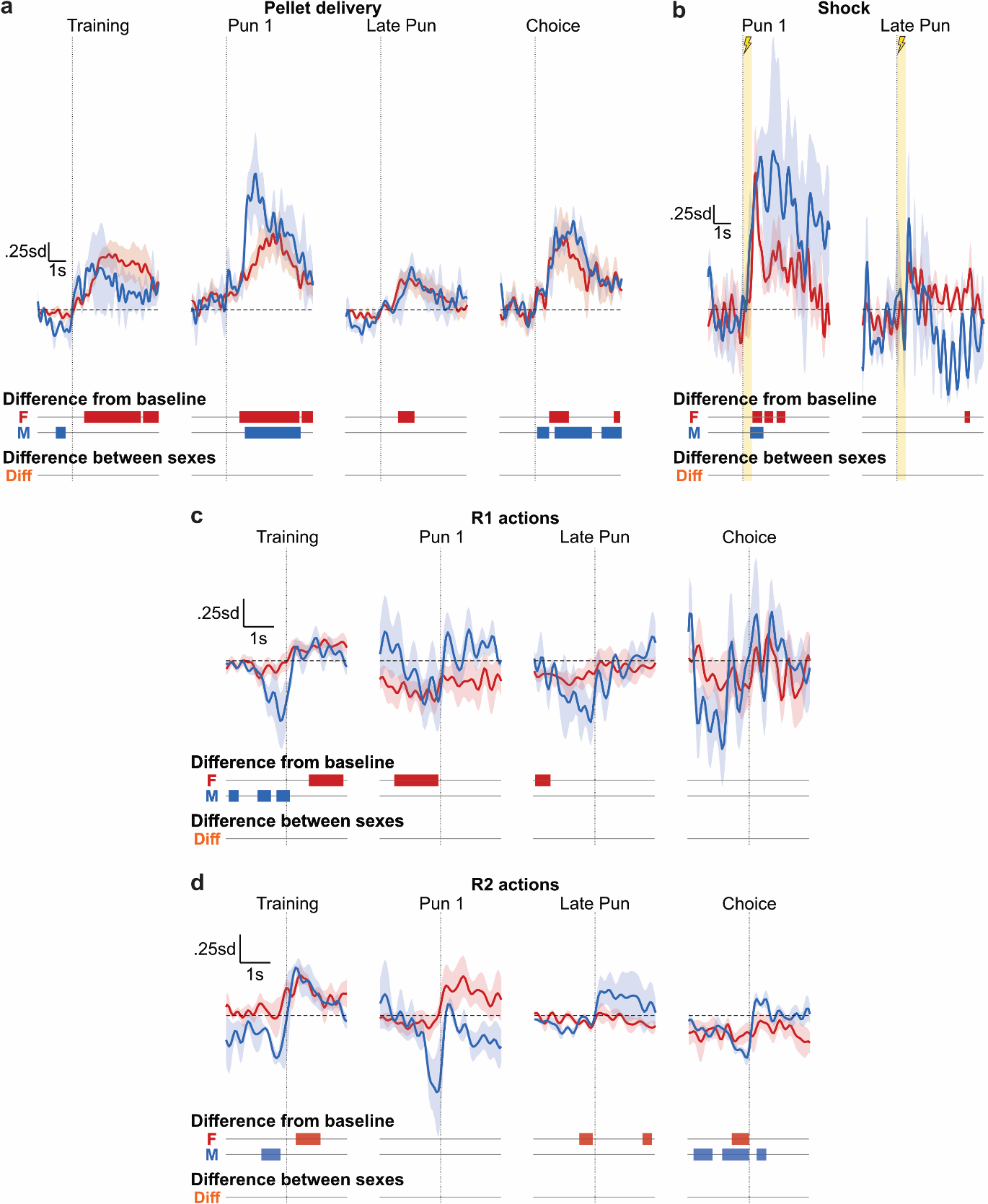
**

**Figure S4. Event-related GABA input to VTA_DA_ by sex. [A-D]** Mean ± SEM of subject-averaged iGABASnFR signals around pellet delivery (**[A]**), footshock delivery (**[B]**), R1 actions alone (**[C]**), and R2 actions alone (**[D]**) for female (F; *n*=7) vs. male (M; *n*=3) subjects across lever training, early punishment (Pun 1 [1^st^ session]), late punishment (sessions 4+), and choice sessions. Vertical dashed lines indicate event onset (yellow highlighted area indicates shock duration). Horizontal dotted line indicates pre-event baseline. Bars at the bottom indicate when peri-event signals significantly deviated from baseline (female = red bars; male = blue bars), and when females vs. males significantly differed from each other (orange bars). No significant sex differences were observed.

**
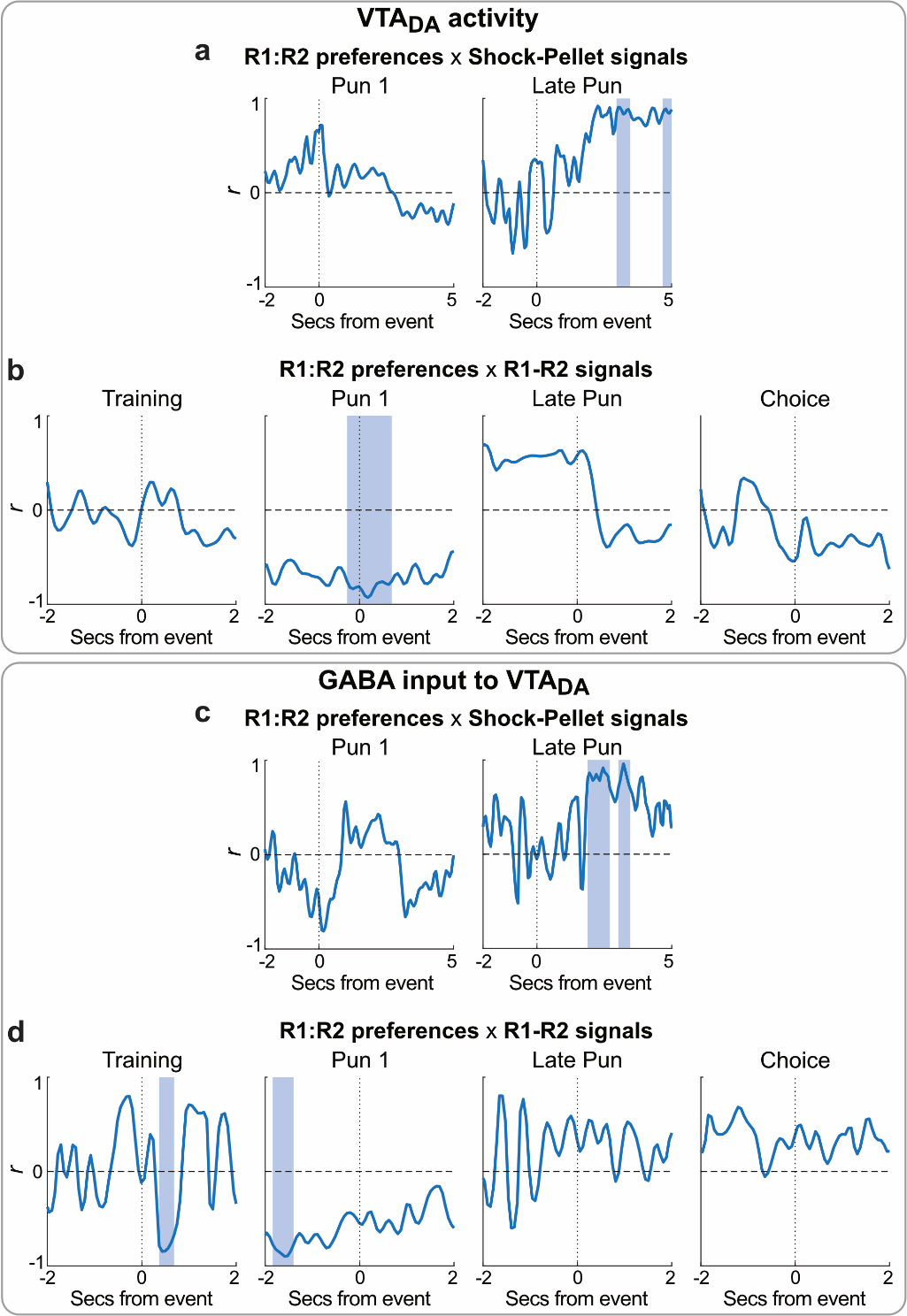
**

**Figure S5. Relationship between event-related signals and behavior.** Correlations between response bias (R1:R2 ratio) and GCaMP (**[A-B];** *n*=7) or iGABASnFR (**[C-D]**; *n*=10) signal bias around outcome/action onset across sessions. Shaded regions indicate when *p*<.05 for >1/3sec (low-pass filter window). **[A]** Correlation between response bias and outcome-related GCaMP signal bias. No significant relationship was observed across the event window for early punishment. In late punishment, increased Shock-Pellet activity was positively correlated with R1 preference; greater appetitive coding bias was associated with greater preference for R2. **[B]** Correlation between response bias and action-related GCaMP signal bias. No significant relationships were observed across sessions except for early punishment, where increased activity around R1 responses was associated with increased avoidance of R1. **[C]** Correlation between response bias and outcome-related iGABASnFR signal bias. No significant relationship was observed for early punishment. In late punishment, increased Shock-Pellet activity was positively correlated with R1 preference; reduced aversive coding bias was associated with greater preference for R2. **[D]** Correlation between response bias and action-related iGABASnFR signal bias. During pre-punishment training, increased activity following R2 responses was associated with increased R2 preference. During early punishment, increased activity prior to R2 responses was associated with increased R2 preference. No significant correlations were observed for late punishment and choice.

**
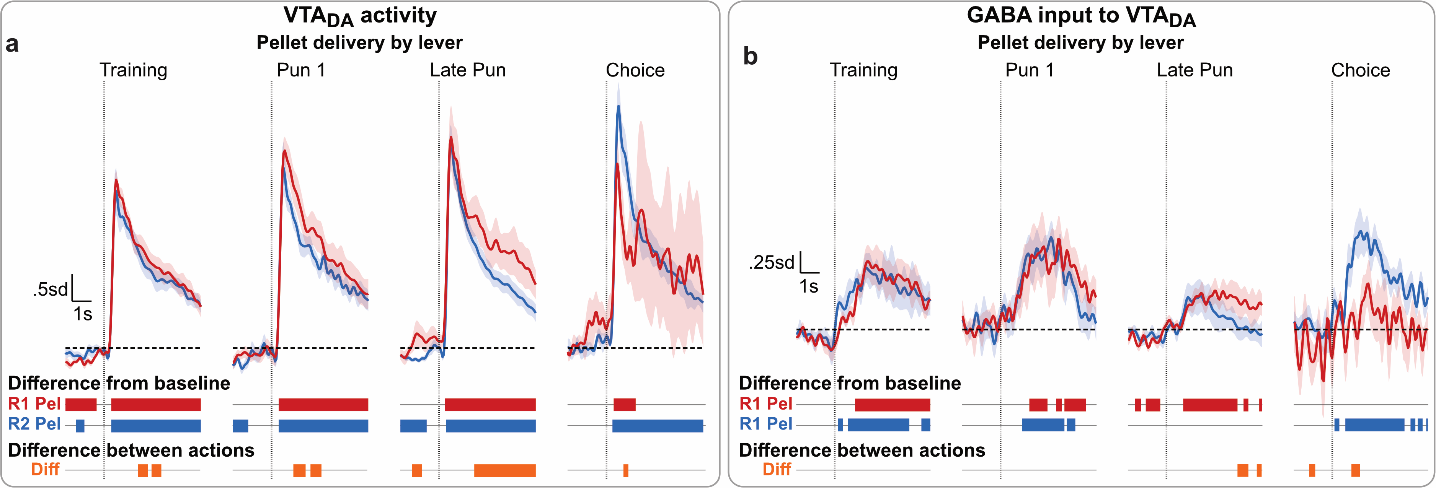
**

**Figure S6. Pellet delivery signals by lever.** Mean ± SEM of subject-averaged GCaMP (**[A]**; *n*=7) and iGABASnFR (**[B]**; *n*=10) signals around R1-elicited (red) vs. R2-elicited (blue) pellet deliveries for lever training, early punishment (Pun 1 [1^st^ session]), late punishment (sessions 4+), and choice. Vertical dashed lines indicate event onset. Horizontal dotted line indicates pre-event baseline. Bars at the bottom indicate when peri-event signals significantly deviated from baseline, and when pellet signals significantly differed from each other (orange bars).

**
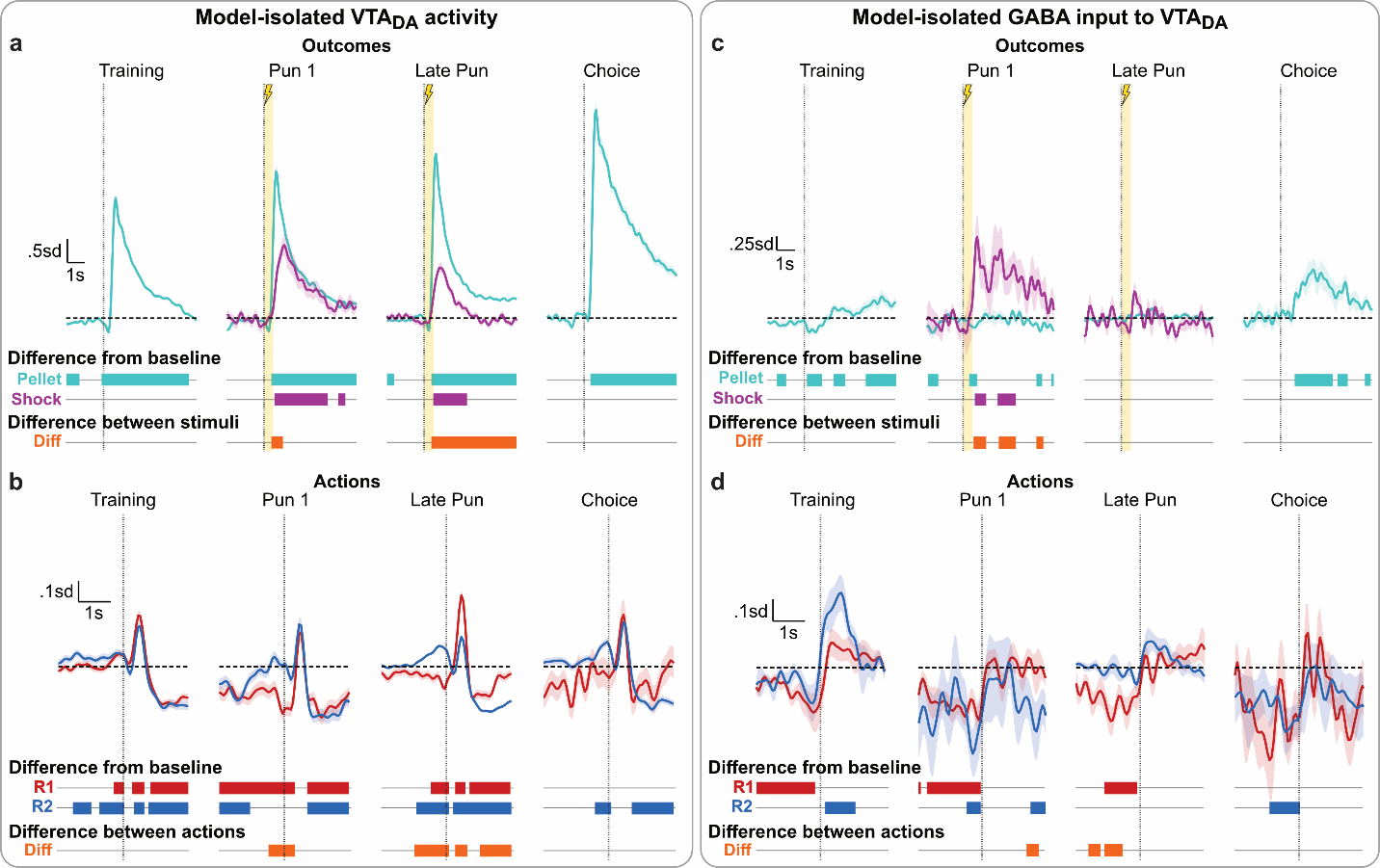
**

**Figure S7. General Linear Modelling of event-related signals. [A-D]** Mean ± SEM of isolated outcome- and action-related signals derived from General Linear Modelling. **[A]** As found with conventional analyses (Figure 1), there were larger spikes in VTA_DA_ calcium activity to pellet delivery (teal) than aversive footshock [purple]). **[B]** VTA_DA_ activity to punished R1 actions became dissociated from unpunished R2 actions as punishment was learned. **[C]** As with conventional analyses, there was a transient increase in GABA input to VTA_DA_ to aversive footshock [purple]) in initial (Pun1) but not later punishment. **[D]** Unlike conventional analyses, a significant difference in GABA input to VTA_DA_ around punished R1 vs. unpunished R2 actions was detected during punishment sessions, although differences were not robust across sessions.

**
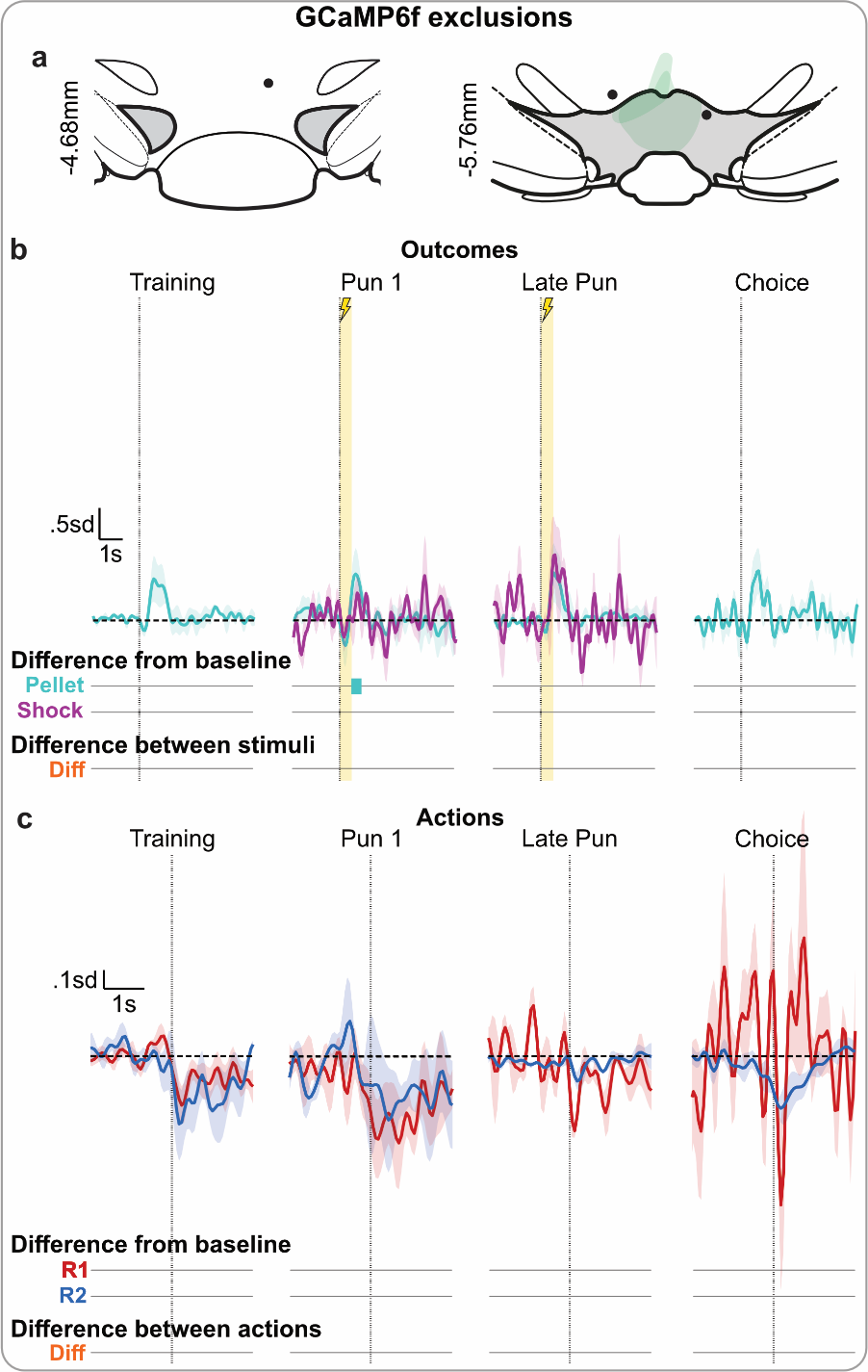
**

**Figure S8. Analysis of photometry signals from excluded subjects. [A]** Placement map for subjects with misplaced fiber implant (*n*=1) or poor GCaMP6f expression (*n*=2) (*N*=3). **[B-C]** Mean ± SEM of subject-averaged signals around **[B]** response-elicited outcomes (pellet delivery [teal] vs. footshock [purple]) and **[C]** actions (R1 vs. R2 action alone; no outcomes delivered) during lever training, early punishment (Pun 1 [1^st^ session]), late punishment (sessions 4+), and choice sessions. Vertical dashed lines indicate event onset (yellow area indicates shock duration). Horizontal dotted line indicates pre-event baseline. Bars at the bottom indicate when peri-event signals significantly deviated from baseline, and when signals significantly differed from each other (orange). Recordings without correct fiber placement and adequate GCaMP6f expression did not produce the robust peri-event signals reported in the main text, indicating photometry findings are not easily attributable to background and/or movement-related artifacts.


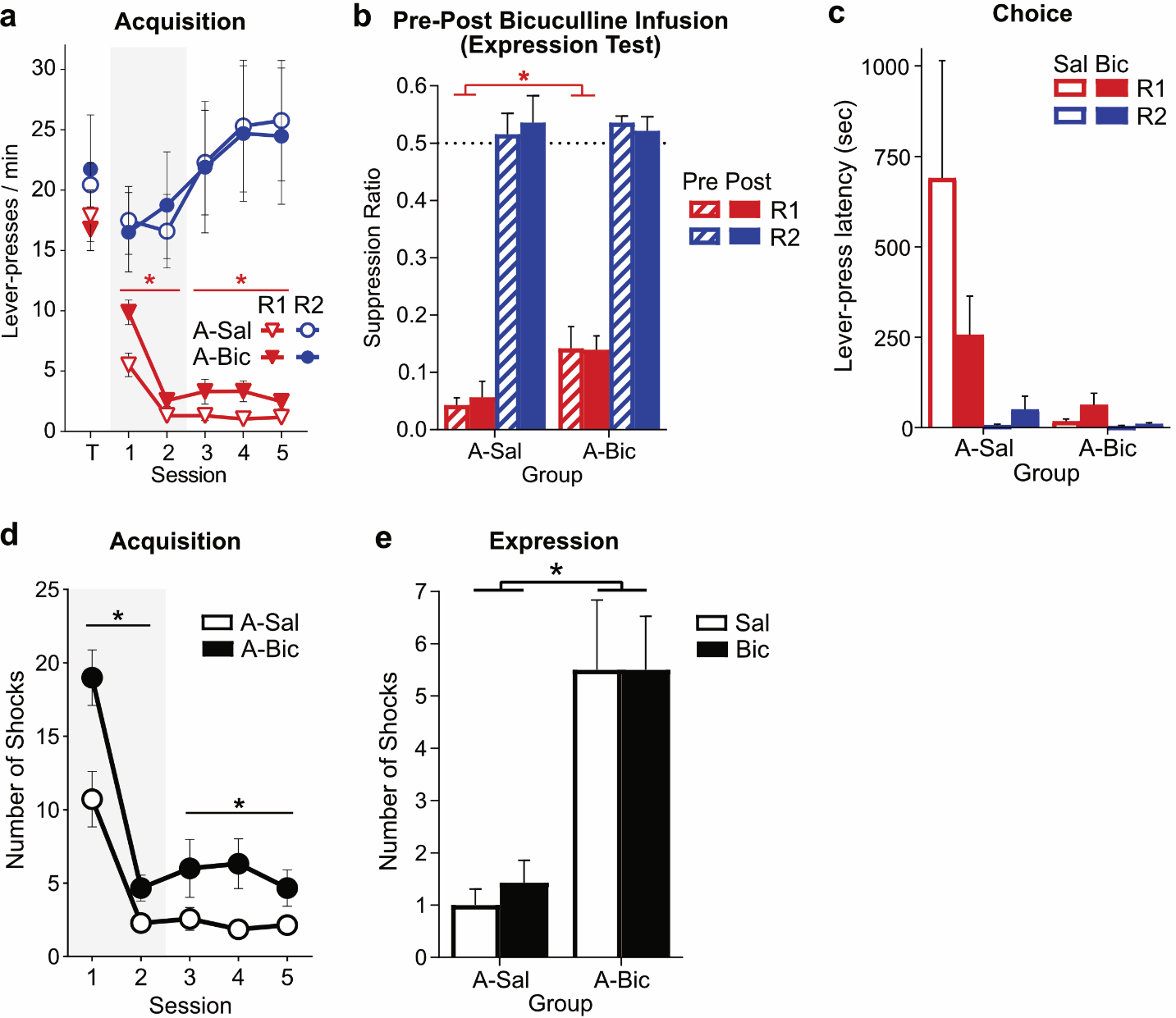


**Figure S9. Supplementary results for Experiment 2 (VTA GABA_A_ blockade) behavior. [A]** Mean ± SEM lever-press rates for R1 and R2 during last day of training (T) and punishment acquisition sessions. A-Bic vs. A-Sal groups received bicuculline (Bic) vs. saline (Sal) (respectively) before the first 2 sessions of punishment (shaded region). A-Bic rats exhibited a persistent deficit in punishment avoidance. **[B]** Mean ± SEM suppression ratios for R1 and R2 lever-presses during the punishment session before (Pre) and after (Post) Bic expression test. Bic during expression had no effect on subsequent punishment avoidance in either A-Sal or A-Bic group. **p*<.05. **[C]** Mean ± SEM initial lever-press latencies during 30min choice tests. Subjects were slower to press R1 than R2 (lever: *F*_(1,11)_=6.46, *p*=.027). There was a trend towards faster press latencies for A-Bic animals relative to A-Sal rats (group: *F*_(1,11)_=4.69, *p*=.053), with a trend towards this depending on lever (group*lever: *F*_(1,11)_=4.78, *p*=.051). **[D]** Mean ± SEM number of shocks delivered across punishment acquisition sessions. A-Bic rats were shocked, on average, significantly more than A-Sal rats both during infusion (group: *F*_(1,11)_=10.60, *p*=.008) and post-infusion (group: *F*_(1,11)_=6.14, *p*=.031) sessions. **[E]** Mean ± SEM number of shocks delivered during punishment expression tests by acquisition group. A-Bic subjects received significantly more shocks than A-Sal subjects (group: *F*_(1,11)_=16.15, *p*=.002). Acute bicuculline infusions during expression had no effect on this (drug: *F*_(1,11)_=0.206, *p*=.659; drug*group: *F*_(1,11)_=0.206, *p*=.659).


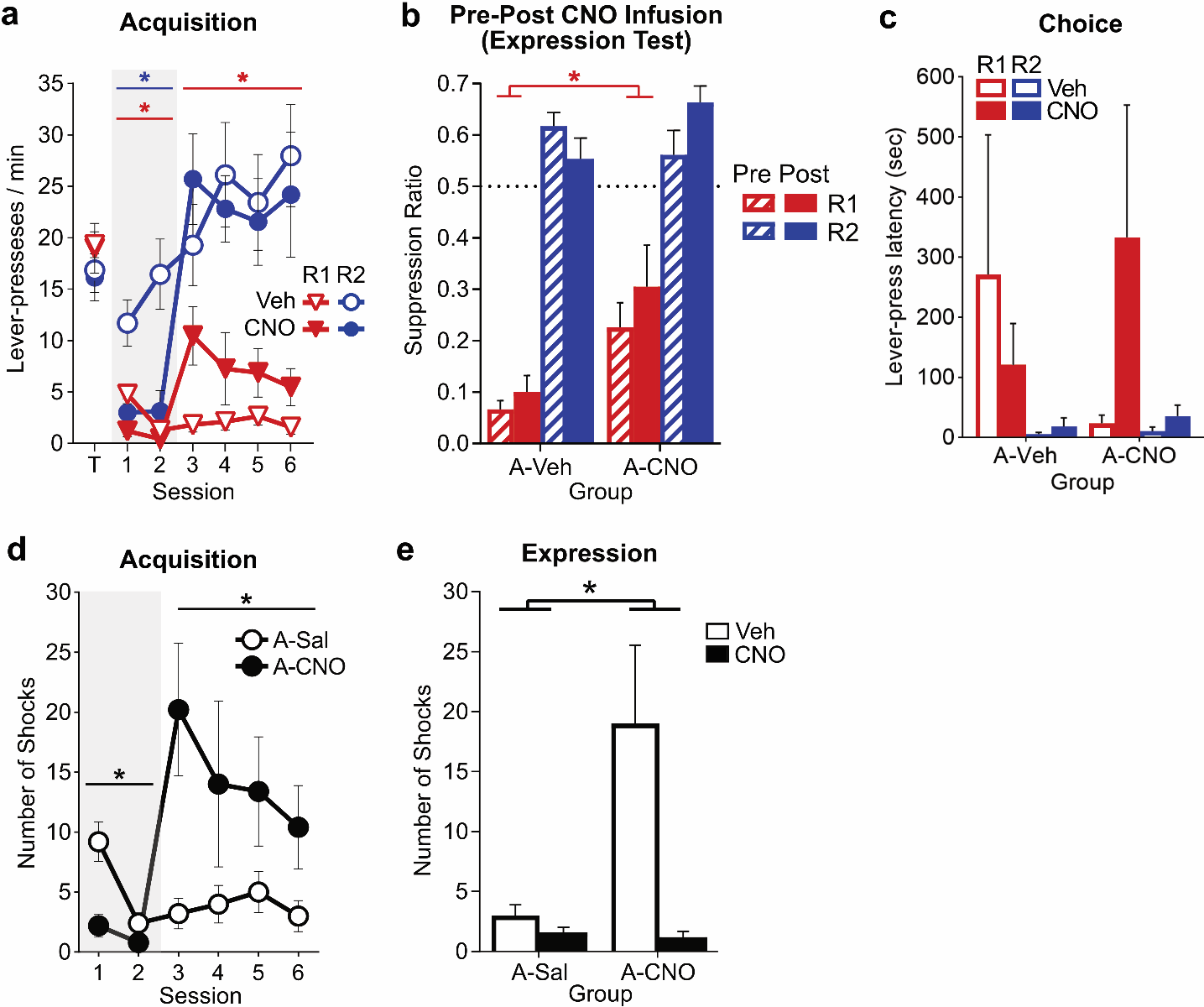


**Figure S10. Supplementary results for chemogenetic activation of VTA_DA_ neurons during punishment. [A]** Mean ± SEM lever-press rates for R1 and R2 during last day of training (T) and punishment acquisition sessions. A-CNO vs. A-Veh groups received CNO vs. vehicle (respectively) before the first 2 sessions of punishment (shaded region). **[B]** Mean ± SEM suppression ratios for R1 and R2 lever-presses during the punishment session before (Pre) and after (Post) CNO expression test. **p*<.05. **[C]** Mean ± SEM initial lever-press latencies during 20min choice tests. There was a non-significant trend towards faster pressing of R1 relative to R2 (lever: *F*_(1,8)_=4.68, *p*=.063). In line with lever suppression ratios, there was a trend towards slower latencies to respond on levers after CNO injections except for A-Veh R1 responses. However, these trends were not significant (drug/group/lever interactions: all *F*_(1,8)_≤1.83, *p*≥.214). **[D]** Mean ± SEM number of shocks delivered across punishment acquisition sessions. A-Veh rats incurred significantly more shocks than A-CNO rats during infusion days (group: *F*_(1,8)_=10.33, *p*=.012). Across post-infusion days, A-CNO rats receiving more shocks compared to their A-Veh counterparts (group: *F*_(1,8)_=5.49, *p*=.047). **[E]** Mean ± SEM number of shocks delivered during punishment expression tests by acquisition group. A-Bic rats incurred substantially more shocks than A-Sal rats, particularly following vehicle injections (group: *F*_(1,8)_=5.31, *p*=.050; drug*group: *F*_(1,8)_=6.37, *p*=.036). In correspondence with general decreases in responding, CNO administration reduced numbers of shocks received during Expression CNO tests (drug: *F*_(1,8)_=8.74, *p*=.018).

**
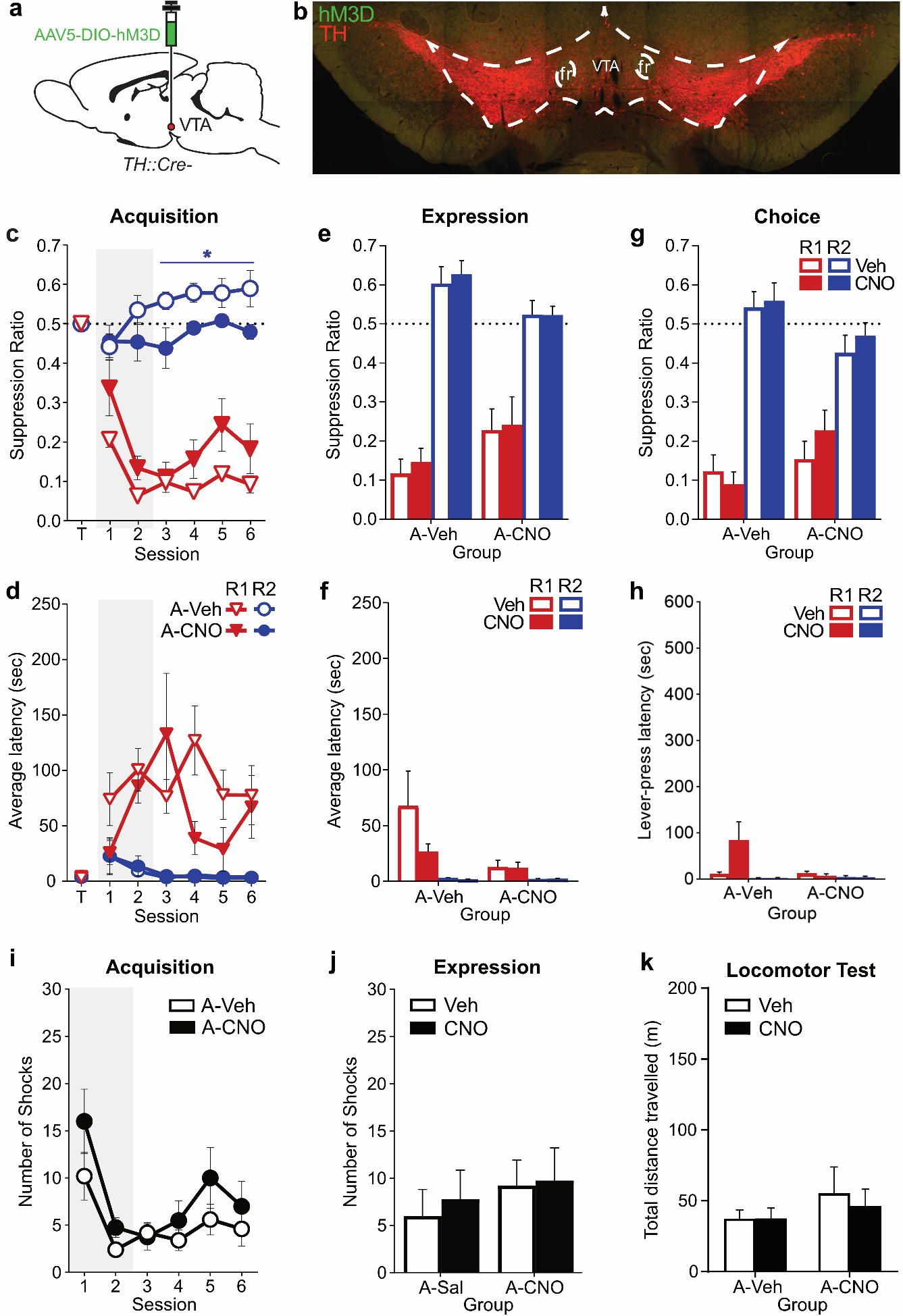
**

**Figure S11. Effects seen in** **TH::Cre- animals during punishment.** **[A]** TH::Cre- rats received virus encoding Cre-dependent excitatory DREADD hm3D bilaterally into the VTA. Given these animals do not express Cre in any cells, no hm3D expression was expected; these animals serve as control comparisons for the TH::Cre+ experiment reported in main text/figures. **[B]** Example hm3D and TH expression in TH::Cre- animal. No hm3D expression was observed. **[C]** Mean ± SEM lever suppression ratios by acquisition group (A-Veh [*n*=5], A-CNO [*n*=4]) across last day of training (T) and punishment acquisition sessions. Grey shaded area indicates injection sessions. In contrast to the effects of CNO in TH::Cre+ rats, CNO did not significantly affect behavior during injection sessions (group: *F*_(1,7)_=0.54, *p*=.486) or during subsequent post-injection sessions (group: *F*_(1,7)_=0.07, *p*=.799). There was a significant but uninteresting difference between groups in unpunished responding during these post-injection sessions (*F*_(1,7)_=6.57, *p*=.037). **[D]** Mean ± SEM lever-press latencies by acquisition group across the last day of training (T) and punishment acquisition sessions. Unlike CNO effects in TH::Cre+ animals, CNO did not significantly affect responding in TH::Cre- animals across punishment acquisition (group[injection days]: *F*_(1,7)_=0.84, *p*=.390; group[post-injection days]: *F*_(1,7)_=0.51, *p*=.498). **[E]** Mean ± SEM lever suppression ratios during punishment expression tests. No significant effect of acquisition group was observed (*F*_(1,7)_= 0.01, *p*=.916). **[F]** Mean ± SEM lever-press latencies during punishment expression tests. No significant effects were observed (drug: *F*_(1,7)_=2.05, *p*=.195**;** group *F*_(1,7)_=2.57, *p*=.153). **[G]** Mean ± SEM suppression ratios during choice tests. No significant effects were observed (drug: *F*_(1,7)_=1.66, *p*=.239**;** group *F*_(1,7)_=0.05, *p*=.825). **[H]** Mean ± SEM initial lever-press latencies during 20min choice tests. There was a non-significant trend towards faster pressing of R1 relative to R2 (lever: *F*_(1,7)_=5.15, *p*=.057). There were no effects of injection or acquisition group (drug: *F*_(1,7)_=2.18, *p*=.183; group: *F*_(1,7)_=2.15, *p*=.146; drug/group/lever interactions: all *F*_(1,7)_≤2.99, *p*≥.122). **[I]** Mean ± SEM number of shocks delivered across punishment acquisition sessions. No significant effect of acquisition group was observed during injection (group: *F*_(1,7)_=2.48, *p*=.159) or post-injection sessions (group: *F*_(1,7)_=0.69, *p*=.435). **[J]** Mean ± SEM number of shocks delivered during punishment expression tests by acquisition group. No effect of acquisition group (group: *F*_(1,7)_=0.37, *p*=.561) or acute CNO (session: *F*_(1,7)_= 3.775, *p*=.093; session*group: *F*_(1,7)_= 1.206, *p*=.308) was observed. **[K]** Mean ± SEM distance travelled during locomotor tests. Unlike TH::Cre+ subjects, no significant effects were observed (drug: *F*_(1,6)_=0.78, *p*=.411**,** group: *F*_(1,7)_=0.94, *p*=.370).
